# Standardization of preclinical methodologies for discovery and validation of circulating microRNA biomarkers for post-traumatic epileptogenesis – Lessons learned from the EpiBioS4Rx Project 1

**DOI:** 10.1016/j.eplepsyres.2025.107667

**Published:** 2025-09-19

**Authors:** Noora Puhakka, Mette Heiskanen, Xavier Ekolle Ndode-Ekane, Idrish Ali, Cesar Santana-Gomez, Shalini Das Gupta, Meheli Banerjee, Pedro Andrade, Riikka Immonen, Pablo Casillas-Espinosa, Gregory Smith, Rhys D. Brady, Juliana Silva, Emma Braine, Matthew R. Hudson, Glen R. Yamakawa, Nigel C. Jones, Sandy R. Shultz, Neil G. Harris, David K. Wright, Olli Gröhn, Richard Staba, Terence J. O’Brien, Asla Pitkänen

**Affiliations:** a A. I. Virtanen Institute for Molecular Sciences, University of Eastern Finland, PO Box 1627, Kuopio FI-70211, Finland; b Department of Neuroscience, Monash University, Melbourne, Australia; c Department of Neurology, Alfred Health, Melbourne, Australia; d Department of Medicine, The Royal Melbourne Hospital, The University of Melbourne, Parkville, Australia; e Department of Neurology, David Geffen School of Medicine at UCLA, Los Angeles, CA, 90095, United States; f UCLA Brain Injury Research Center & Department of Neurosurgery, David Geffen School of Medicine at UCLA, Los Angeles, CA, 90095, United States

**Keywords:** Harmonization, Lateral fluid-percussion, MicroRNA, Plasma, Preclinical, Post-traumatic epilepsy

## Abstract

**Objective:**

To analyze the success of harmonization and standardization of plasma miRNA biomarker discovery and validation for post-traumatic epilepsy (PTE) in the EpiBioS4Rx international multicenter project.

**Methods:**

Adult male Sprague-Dawley rats were randomized to lateral fluid-percussion-induced traumatic brain injury (TBI) or sham operation at three study sites (Finland, Australia, USA). Video-electroencephalogram (vEEG) was performed in the 7th post-injury month to detect spontaneous seizures. Tail vein plasma was collected at baseline and 48 h after TBI for microRNA (miRNA) analysis. Common data elements were generated to document and monitor pre-analytic activities, including housing conditions, post-injury care, blood sampling, plasma preparation, plasma quality, storage, and shipping. miRNA analysis was performed using droplet digital PCR (ddPCR) at one study site (Finland) with on-site standardized procedures.

**Results:**

The 2-day miRNA levels were successfully measured in 85 % (209/245) of the rats included in the final analysis cohort. Exclusions were related to small sample volume, hemolysis, and failed RNA extraction for ddPCR. Most of the pre-analytical factors leading to sample exclusions were related to non-optimal plasma pipetting. We also recognized gaps in data entry and monitoring of personnel training.

**Conclusions:**

Our study demonstrates that conducting a successful plasma miRNA biomarker analysis requires procedural harmonization between laboratories, protocol standardization, inclusion and analysis of quality controls, training of researchers, and continuous monitoring of adherence to pre-agreed protocols.

## 1. Introduction

Worldwide, over 50 million people have epilepsy (https://www.who.int/news-room/fact-sheets/detail/epilepsy). About 10–15 % of structural epilepsies, and 5 % of all epilepsies, are caused by traumatic brain injury (TBI)(Hauser et al., 1993; Herman, 2002). Even though over 20 favorable preclinical proof-of-concept studies have reported some disease-modifying effects in models of post-traumatic epileptogenesis, no clinically proven interventions are available for at-risk persons, and their development remains a research priority (Dulla and Pitkänen, 2021; Kelley et al., 2009; Pitkänen et al., 2019a). One major obstacle to therapy development is the lack of prognostic and predictive biomarkers for epileptogenesis that could be used to stratify populations of TBI patients for antiepileptogenesis trials and serve as surrogate endpoints to reduce study costs, making sufficiently powered clinical trials affordable (Engel et al., 2013; Lapinlampi et al., 2017; O’Brien et al., 2013; Simonato et al., 2014).

The Epilepsy Bioinformatics Study for Antiepileptogenic Therapy (EpiBioS4Rx) is a National Institute of Neurological Disorders and Stroke (NINDS) funded Center-Without-Walls international project (https://epibios.loni.usc.edu/), aiming to facilitate the development of antiepileptogenic therapies to reduce the risk of post-traumatic epilepsy (PTE) after TBI. The preclinical EpiBioS4Rx Project 1 was performed at the three participating study sites (Finland, Australia, USA), and its major aim is to identify clinically translatable prognostic biomarkers such as circulating microRNAs for PTE (Kamnaksh et al., 2019).

Similar to clinical studies, preclinical multi-center biomarker discovery must comply with harmonization of procedures and standardization of sampling and analysis protocols, accurate data collation and sufficient personnel training related to the pre-analytical and analytical factors (Miller et al., 2014). Pre-analytical factors related to analysis of circulating miRNA biomarkers consist of sample collection (*e.g.,* hemolysis), processing (*e.g.,* centrifugation protocol), storage and sample transport which can influence the sample quality, and consequently, the biomarker analysis (Batool et al., 2023; Becker and Lockwood, 2013; Khan et al., 2017). Analytical factors include technical variability due to, for example, RNA extraction method (Meerson and Ploug, 2016; Sourvinou et al., 2013; Wong et al., 2019) or library preparation (Androvic et al., 2022; Baran-Gale et al., 2015; Wong et al., 2019). Furthermore, bioinformatics methods and parameters used in the downstream analysis of the sequencing data can influence the results (Bisgin et al., 2018; Metpally et al., 2013; Tam et al., 2015). Selection of normalization strategy (*e.g.,* endogenous reference miRNA or exogenous spike-in) can affect the output related to the quantification in miRNA expression (Binderup et al., 2018; Faraldi et al., 2019). Further, in the analysis of large sample numbers with multiple PCR plates, between-runs variation can affect the absolute values and batch comparisons (Ruijter et al., 2015). Several large-scale preclinical plasma biomarker studies for brain diseases have been performed or are ongoing (Kochanek et al., 2016; Radabaugh et al., 2025; Santorelli et al., 2024; van Vliet et al., 2024; Wanner et al., 2025) and both the TBI and epilepsy fields are making significant progress in generating common data elements (CDEs) for preclinical research (Harte-Hargrove et al., 2017; Lapinlampi et al., 2017; Laplaca et al., 2021; Smith et al., 2015). However, there is still little experience in the success of harmonization and standardization procedures related to circulating biomarker discovery and validation.

To perform a statistically powered preclinical biomarker discovery, EpiBioS4Rx Project 1 made a major attempt to harmonize the production of the rat PTE model induced with lateral fluid-percussion injury (FPI) to generate a well-phenotyped pool of animals for biomarker discovery and the collection of plasma at the three participating study sites. Moreover, significant efforts were made to standardize the miRNA analysis (Kamnaksh et al., 2019; Ndode-Ekane et al., 2024b). To judge the success of the harmonization and standardization, we analyzed the quality control data collected during the biomarker discovery and validation phases. Our efforts to harmonize study protocols at pre-analytical and analytical phases were successful, and analytical factors did not compromise the reliability of the miRNA biomarker analysis. However, we also recognized some weaknesses in our approaches, which can be improved to optimize protocols for preclinical discovery of circulating biomarkers.

## 2. Materials and methods

**Fig. 1.**
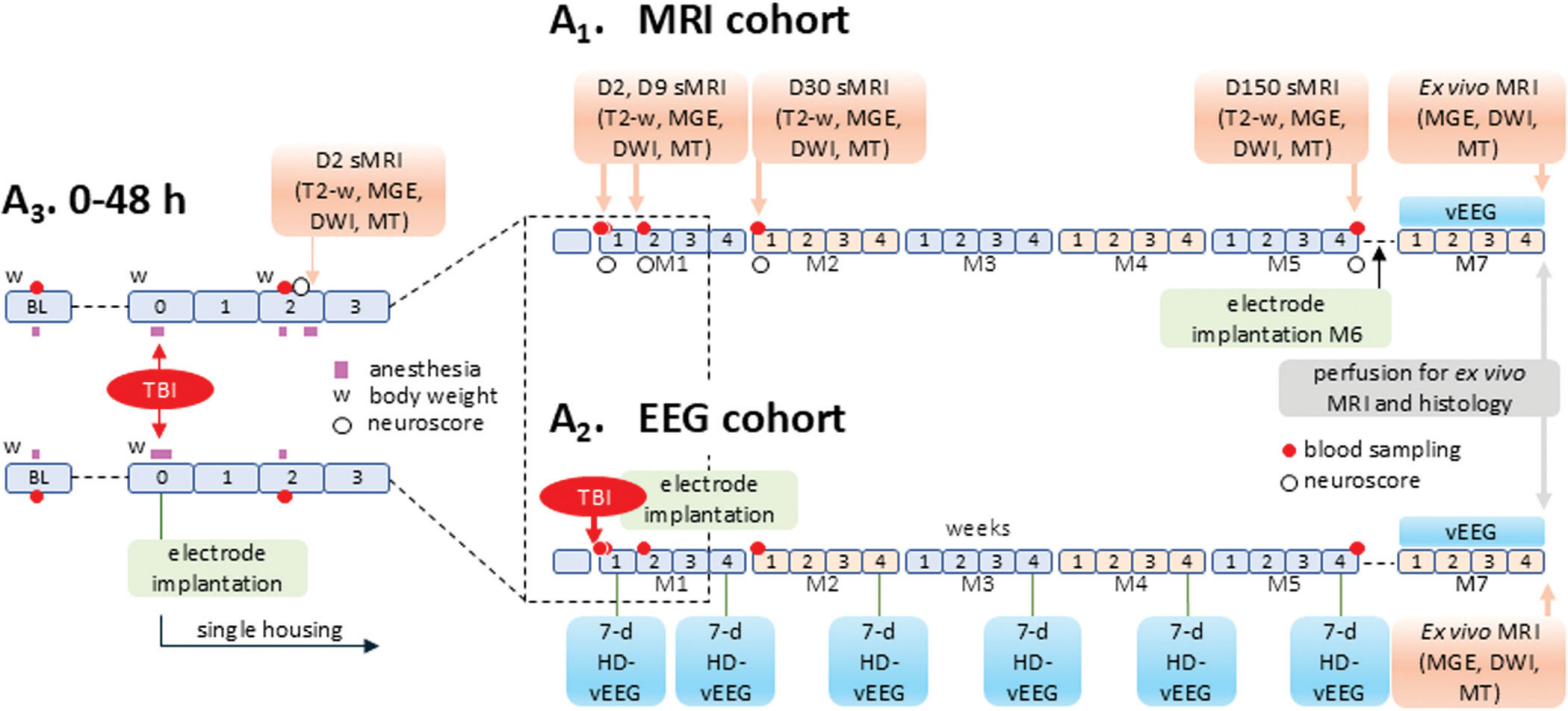
Study design – focus on the first 48 h. The study cohort included rats in the **(A**_**1**_) MRI cohort and **(A**_**2**_) EEG cohort. For details, see Ndode-Ekane et al. (2024) and Fig. 2. **(A**_**3**_) Micro RNAs (miRNAs) were analyzed in plasma samples collected on day (D) 2 (48 h) post-TBI or sham-operation. Also, baseline (naive) plasma samples, which had been collected on the week preceding craniectomy or TBI were included in the analysis. On D2, blood sampling was performed before the composite neuroscore test or MRI imaging. Note that animals experienced a total of 3 anesthesia sessions before completing the D2 blood sampling (baseline blood sampling, D0 craniectomy or TBI w/wo electrode implantation, and D2 blood sampling). Rats were single-housed after surgery. ***Abbreviations***: BL, baseline; DWI, diffusion weighted imaging for diffusion tensor imaging; HD, high-density; M, month; MGE, multiecho gradient echo imaging; MT, magnetization transfer imaging; sMRI, structural magnetic resonance imaging; T2-w, T2-weighted imaging; TBI, traumatic brain injury; vEEG, video-electroencephalogram.

Study design and timing of sample collection are summarized in Fig. 1. To perform a statistically powered plasma biomarker discovery study in a large animal cohort, EpiBioS4Rx Project 1 was performed in three sites located in Finland (University of Eastern Finland, Kuopio), Australia (Monash University, Melbourne), and the USA (David Geffen School of Medicine at University of California, Los Angeles; UCLA). All study sites implemented harmonized protocols for model generation, blood collection, plasma preparation and plasma storage. To reduce variability in miRNA assay, analysis was centralized to the UEF study site that used single-site standardized assay protocols.

To maximize the harmonization efficiency, details of procedures, including the selection of materials and equipment for blood collection and plasma preparation, were agreed upon in a series of teleconferences before initiating the project. An *interim* analysis of the success in procedural harmonization was conducted for the first cohort of animals (Casillas-Espinosa et al., 2019; Ciszek et al., 2019; Immonen et al., 2019; Kamnaksh et al., 2019; Ndode-Ekane et al., 2019; Pitkänen et al., 2019b; Santana-Gomez et al., 2019), and an attempt was made to fix any inconsistencies identified.

### 2.1. Ethics

#### 2.1.1. UEF

The animal procedures were approved by the Animal Ethics Committee of the Provincial Government of Southern Finland. All animal procedures were performed by competent personnel according to European Union legislation concerning the use of animals for scientific purposes (Directive 2010/63/EU).

#### 2.1.2. Monash

All animal procedures were approved by the Florey Animal Ethics Committee (ethics number 17–014 UM) at the University of Melbourne and by the Alfred Medical Research & Education Precinct Animal Ethics Committee (E/1799/2018/M) at Monash University.

#### 2.1.3. UCLA

All animal procedures were approved by the University of California Los Angeles Institutional Animal Care and Use Committee (protocol 2000–153–61 A).

### 2.2. Animal cohort

**Fig. 2.**
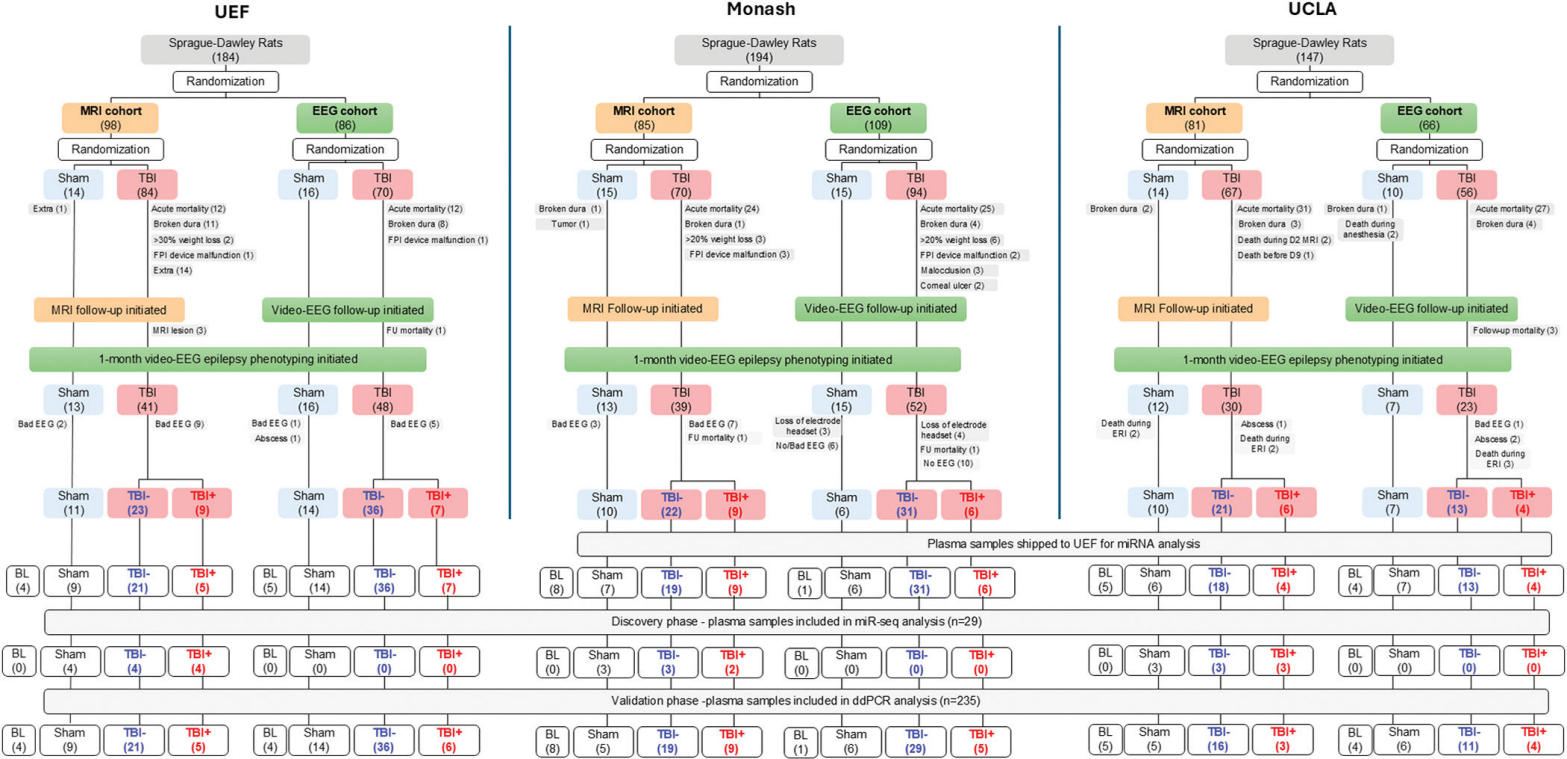
Study flow and success of blood sampling on D2 for miRNA analysis at different study sites. UEF randomized 184 rats, Monash 194 rats and in UCLA 147 rats into the EpiBioS4Rx Project 1. The final vEEG-phenotyped analysis cohort included 100 rats in UEF (43 in MRI and 57 in EEG cohort), 84 in Monash (41 MRI, 43 EEG) and 61 in UCLA (37 MRI, 24 EEG). Causes of exclusions are shown in grey shading. On D2, plasma was sampled from 92 % (92/100) of rats in UEF (35/43 MRI, 57/57 EEG), from 93 % (78/84) of rats in Monash (35/41 MRI, 43/43 EEG) and from 85 % (52/61) of rats in UCLA (28/37 MRI, 24/24 EEG). Note that the use of samples for miRNA biomarker discovery by using miR-seq reduced the sample numbers available for validation by droplet digital PCR (ddPCR). After quality controls, miRNA analysis using ddPCR was performed in 91 % (91/100) of rats in UEF, in 87 % (73/84) of rats in Monash and in 74 % (45/61) of rats in UCLA. Thus, miRNA analysis was completed as planned in 85 % (209/245) of the study cohort. The number of animals at each step is in parentheses. ***Abbreviations:*** ddPCR, droplet digital polymerase chain reaction; EEG, electroencephalogram; ERI, electrode re-implantation; FPI, fluid-percussion injury; FU, follow-up; miRNA, micro ribonucleic acid; MRI, magnetic resonance imaging; TBI, traumatic brain injury; TBI−, TBI rats without epilepsy; TBI+ , TBI rats with epilepsy.

Randomization of animals into different treatment groups, exclusions, and sample numbers available for miRNA analysis are shown in Fig. 2.

#### 2.2.1. Model generation

Briefly, severe TBI (anticipated ≤48 h mortality 25 %) was induced using lateral FPI in adult male Sprague-Dawley rats under isoflurane anesthesia as described in detail before (Ndode-Ekane et al., 2024a). Sham-operated experimental controls underwent all surgical procedures without exposure to impact.

Of the injured rats, 22 % developed epilepsy, which was verified using a 1-month-long (24/7) video-EEG monitoring. There was no difference in epilepsy rate between the three study sites, indicating success in procedural harmonization in model generation (Ndode-Ekane et al., 2024a).

#### 2.2.2. miRNA analysis cohort

The video-EEG phenotyped analysis cohort (n = 245) included two sub-cohorts, which were tailored to the needs of magnetic resonance imaging (MRI) or electroencephalogram (EEG) biomarker discovery: MRI cohort (n = 121) and EEG cohort (n = 124). In both cohorts, blood sampling for miRNA biomarker discovery was performed at baseline (before sham-operation or TBI surgery) and then on day (D) 2 (48 h), D9, D30, and D150 after operation.

miRNA analysis was divided into discovery and validation phases. The discovery phase included 30 plasma samples (10 sham, 10 TBI−, 10 TBI+) from the MRI cohort that were used for miRNA-sequencing to reveal the top biomarker candidates for post-traumatic epileptogenesis. Of these, one sample (#2088, TBI+) was excluded after sequencing due to a short duration of video-electroencephalogram (vEEG) monitoring on the 7th post-injury month. The validation phase included 235 samples from both the MRI and EEG cohorts that were used for the ddPCR assay to measure the concentration of top candidate miRNAs.

Samples for additional analyses to assess the harmonization/standardization of methods were obtained from the UEF rat tissue and blood biobank.

### 2.3. Blood sampling and plasma separation

**Fig. 3.**
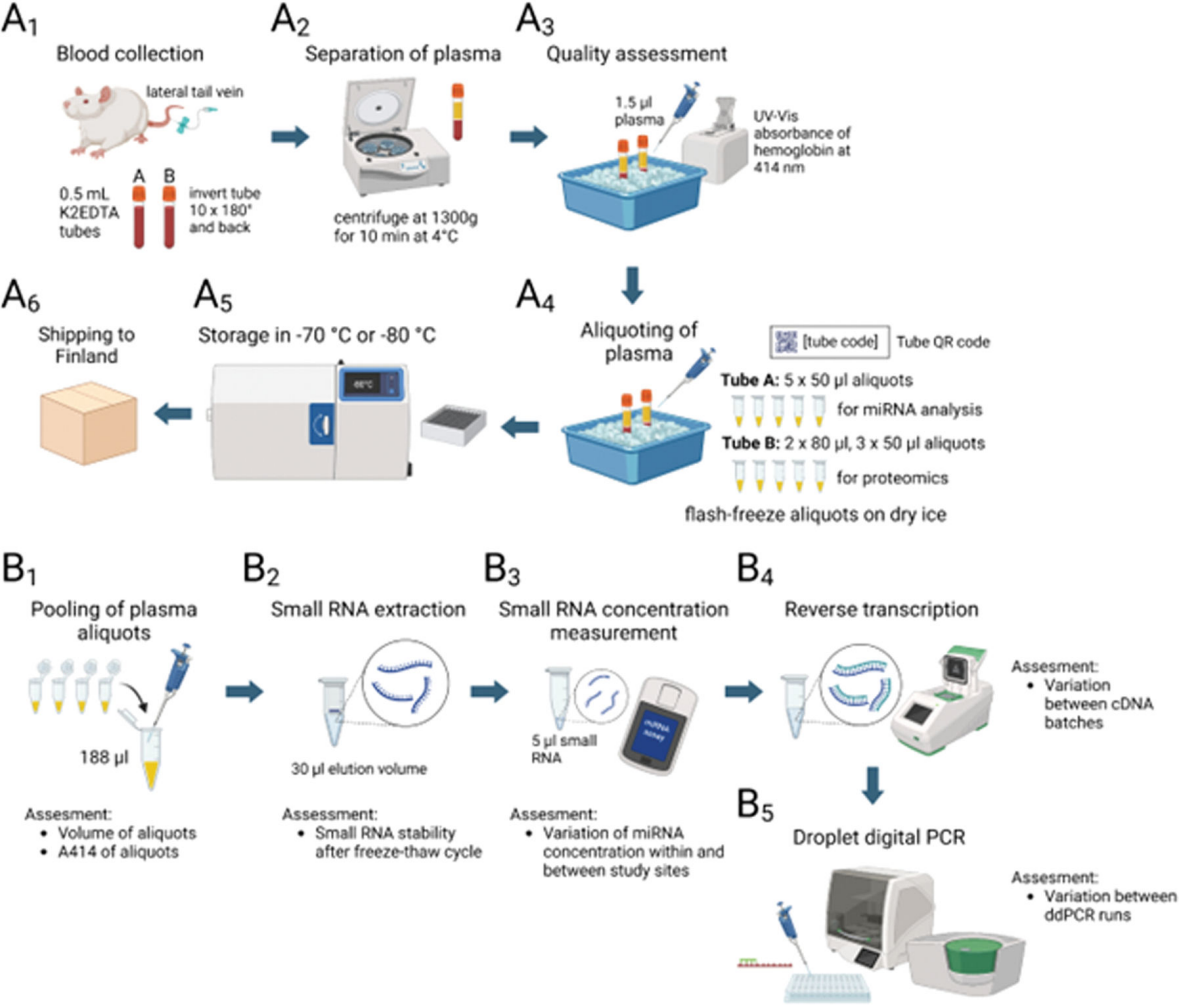
Workflow – Harmonization of blood sampling, plasma preparation and standardization of miRNA analysis. (A_1_) Blood was collected from the lateral tail vein of rats into two 0.5-ml K2EDTA tubes (A and B). **(A**_**2**_) Plasma was separated by centrifugation. **(A**_**3**_) Plasma quality was assessed by measuring the amount of hemolysis by a spectrophotometer at A414 nm. **(A**_**4**_) Then, plasma was aliquoted to 50-μl (miRNA) or 80-μl (proteins) volumes into QR-labeled tubes, which were flash-frozen and **(A**_**5**_) stored at −70°C (UEF) or −80 °C (Monash, UCLA). **(A**_**6**_) After completing the epilepsy phenotyping, frozen samples were shipped to UEF for miRNA analysis. **(B**_**1**_) Then, 47 μl of plasma from four 50-μl aliquots were pooled together to acquire 188 μl plasma for **(B**_**2**_) small RNA extraction. **(B**_**3**_) Small RNA concentration was measured from a 5 μl of 30-μl RNA elute. **(B**_**4**_) RNA in the remaining elute (25 μl) was reverse transcribed to cDNA. Finally, **(B**_**5**_) miRNA expression levels were analyzed using droplet digital PCR (ddPCR).

Next, we describe the protocols applied in blood sampling and sample processing (Fig. 3). Details of the materials used at different study sites and animal conditions that could affect blood chemistry are summarized in Table 1.

#### 2.3.1. Tail vein plasma

Rats were lightly anesthetized with 5 % isoflurane using a nose cone. A 23 G butterfly needle (no heparin) was inserted into the lateral tail vein according to a 3Rs published protocol (Figure 3A_1_; https://nc3rs.org.uk/3rs-resources/blood-sampling/blood-sampling-rat; (Kamnaksh et al., 2019; van Vliet et al., 2017). Tail vein blood (1 ml) was collected into two 500 μl K2EDTA-tubes and mixed by turning the tube 180° and back 10 times. Immediately thereafter, blood samples were centrifuged at 1300 g for 10 min at 4 °C. Supernatant was carefully pipetted, avoiding touching the cellular layer, into 50-μl or 80-μl aliquots in Eppendorf tubes and stored at −70 °C or 80 °C.

#### 2.3.2. Trunk plasma

In additional control experiments performed at UEF, rats were anesthetized with isoflurane for tail vein sampling and then decapitated. The trunk blood was collected into 2 ml K2EDTA-tubes (#368841, Vacutainer, BD Biosciences, Franklin Lakes, NJ, USA; Karttunen et al., 2019)) and mixed by turning the tube 180° and back 10 times and placed into ice. Centrifugation was performed within 1 h after collection (1300 g, 10 min, 4 °C).

To assess sampling success between the centers, we analyzed (1) the percentage of rats in the analysis cohort with successful blood sampling and (2) the percentage of samples with plasma volume enough for analysis.

### 2.4. Plasma quality

#### 2.4.1. A414 nm

The amount of hemolysis in plasma was determined by measuring the hemoglobin UV–vis absorbance at 414 nm (Nanodrop ND-1000). The first analysis was performed after centrifugation (see Fig. 3A_3_). The second analysis was performed after pooling the plasma of four 50 μl sample tubes for small RNA extraction (Fig. 3B_1_). Plasma samples with an absorbance value > 0.25 were considered of poor quality (Kamnaksh et al., 2019; van Vliet et al., 2017).

#### 2.4.2. miR-23a-3p - miR-452a (dCq)

Samples assigned into the discovery study (10 sham, 10 TBI−, 10 TBI+) were also analyzed by measuring the difference in levels between the reference miR-23a and red blood cell-enriched miR-451a (dCq) using qPCR (Shah et al., 2016). The measurement was performed by Qiagen Genomic Services (Hilden, Germany). A dCq value lower than 5 was considered to indicate no hemolysis in the sample. The dCq value higher than 7 was considered to indicate an increased risk of hemolysis (Shah et al., 2016).

To assess sample quality between the study sites and overall, we analyzed: (1) % of original EDTA A/B tubes with plasma A414 < 0.25 (cut-off), (2) % of pooled aliquots with plasma A414 < 0.25 used for miRNA discovery, (3) % of pooled aliquots with plasma A414 < 0.25 used for small RNA extraction and ddPCR, (4) Association between A414 value (hemolysis) of A and B tubes and weight change (%) over the 48 h preceding the blood sampling, (5) % of samples with miR23a-miR451a difference dCq > 7.

### 2.5. Plasma miRNA analysis

Methodology and results of miRNA analysis have been reported earlier (Heiskanen et al., 2024). Briefly, RNA extraction from the plasma samples (D2 post-TBI time point) followed by small RNA sequencing was conducted by the Qiagen Genomic Services (Hilden, Germany). RNA was extracted from 200 μl of plasma using miRNeasy serum/plasma kit (Qiagen). Small RNA library preparation was conducted with QIASeq miRNA library kit (Qiagen). Single-end sequencing of 75-bp reads was performed at a planned 12 M depth by Illumina NextSeq 550. To check the overall quality, raw fastq files were first inspected with FastQC (v.0.11.3). Then, preprocessing and alignment of the reads to the rat genome were performed in the Qiagen Geneglobe Data Analysis Center (DAC) portal (https://geneglobe.qiagen.com/in/analyze). The processing steps included (i) trimming of the 3’-adapter and low-quality bases with cutadapt, (ii) identification of insert sequences and UMIs (reads with < 16 bp insert sequences or < 10 bp UMI sequences were discarded), (iii) alignment of the processed reads to the rat reference genome RGSC Rnor_6.0 using bowtie. Then, miRNA annotation was performed with miRBase v. 21. Differential expression analysis of miRNAs was conducted with DESeq2 package (v.1.42.0) in RStudio (v. 2023.06.0) using R (v.4.3.1). The small RNA sequencing data can be found from the GeneExpression Omnibus (GEO) under accession No. GSE279767.

Next, we focus on steps that we monitored or should have monitored for standardization of the procedures.

### 2.6. Shipment of plasma samples

The 50-μl aliquots of frozen plasma (4 vials, total 200 μl) were shipped from Monash and UCLA to UEF for miRNA analysis in a controlled dry ice shipping package (−70 °C) by a biopharmaceuticals courier service. On arrival in UEF, the boxes were visually checked to confirm that they were still frozen, and then, transferred to −70°C.

#### 2.6.1. miRNA biomarker discovery using small RNA sequencing

Biomarker discovery was performed from a portion of samples (12 %, 30/246) using small RNA sequencing. The 30 plasma samples (250 μl each) were shipped from UEF to Qiagen Genomic Services (Hilden Germany) in a controlled dry ice shipping package (−70 °C) (Heiskanen et al., 2024). One of the samples was excluded from the analysis as the duration of vEEG during the 7th month of this rat did not comply with the duration required by the study protocol.

To assess sample quality, (1) Qiagen Genomic Services performed analysis of miR23a-miR451a as hemolysis indicator. In addition, we analyzed (2) Raw read counts in the datasets obtained, (3) Different small RNA categories and (4) Raw read counts in samples from different sites.

#### 2.6.2. miRNA biomarker validation using ddPCR

##### 2.6.2.1. RNA extraction.

###### EpiBioS4Rx validation cohort.

For miRNA extraction (Fig. 3B_2_), four plasma aliquots (50 μl) were thawed on ice, and 47 μl of each aliquot were pooled to obtain a total of 188 μl plasma. If the total volume of plasma acquired from the aliquots was < 188 μl, all available plasma was pooled, and the final volume was recorded.

###### RNA extraction protocol.

MicroRNA was extracted from the pooled plasma samples using miRNeasy Mini Kit (#217004, QIAGEN) according to the manufacturer’s protocol. For plasma volumes < 188 μl, the reagent volumes in the extraction protocol were adjusted accordingly. The final elution volume for all samples was 30 μl. One 6 μl aliquot of extracted RNA was stored in a separate 0.5 ml Eppendorf tube for measurements of small RNA concentration. Extracted RNA samples were stored in −70 °C.

To assess procedural quality and inter-site differences at validation phase, we analyzed (1) Plasma volume per aliquot of samples at different study sites (50 μl expected), (2) Plasma volume available for RNA extraction (188 μl expected), (3) Effect of training of the researcher on the quality of RNA extraction, (4) Total miRNA concentration, (5) Effect of hemolysis to total miRNA concentration, (6) Effect of post-TBI weight loss on total miRNA concentration.

##### 2.6.2.2. Small RNA concentration.

Small RNA concentration in each sample was measured from 5 μl of extracted RNA using Qubit microRNA Assay Kit (#Q32880, Thermo Fisher Scientific) with DeNovix DS-11 FX Fluorometer (DeNovix Inc., Wilmington, DE, USA).

##### 2.6.2.3. Reverse transcription.

Total RNA was transcribed to cDNA using the miRCURY LNA RT kit (#339340, QIAGEN). The total reaction volume was 10 μl or 20 μl, depending on the sample (2 μl or 4 μl template RNA in each reaction). UniSp6 spike-in (miRCURY LNA RT Kit, #339340) was added in each reverse transcription reaction according to the RT kit’s protocol. The temperature cycling protocol used: incubation for 60 min at 42 °C followed by incubation for 5 min at 95 °C to inactivate the reverse transcriptase, and immediately thereafter, cooling of the sample to 4 °C (T100 Thermal Cycler, #1861096, BIO-RAD). The cDNA samples were stored at −20 °C until further analysis.

### 2.7. Selection of reference miRNAs

#### 2.7.1. Selection of reference genes

miR-28–3p was selected as a reference gene for expression normalization based on our previous NormFinder analyses (Gupta et al., 2021; Heiskanen et al., 2023).Also, in the theEpiBios4Rx material, miR-28–3p was the most stable miRNA across the sequencing samples on D2 post-TBI (Heiskanen et al., 2024).

#### 2.7.2. Data normalization

Data were normalized to miR-28–3p using the formula 2 −ΔCt. Results were plotted and statistically analyzed by GraphPad Prism 8 software (GraphPad Software, San Diego, CA, USA).

### 2.8. ddPCR

#### Primer testing.

For all miRNAs, cDNA was diluted 1:10 in nuclease-free water.

#### Sample analysis.

The ddPCR analysis included samples from a total of 164 TBI rats and 45 sham-operated experimental controls. In addition, 26 baseline (BL) samples collected before the TBI or sham operation were included as naïve control samples.

The ddPCR analysis was conducted in two parts: miR-434–3p, miR-183–3p and miR-323–3p were analyzed first (round 1, May 2021 to August 2021), and miR-9a-3p, miR-124–3p, miR-132–3p, and miR-212–3p were analyzed about 7–12 months later (round 2, March 2022 to May 2022). On both rounds, the order of samples on the plates was randomized. The cDNA synthesis on the second ddPCR analysis round was performed from the same RNA samples that were used in round 1.

The master mix for one ddPCR reaction (reaction volume 20 μl) contained 10 μl of 2 X QX200 ddPCR EvaGreen SuperMix (#1864034, BIO-RAD), 1.0 μl nuclease-free water, and 1.0 μl miRCURY LNA PCR assay (QIAGEN). Each reaction contained 8 μl of the cDNA template, which had been diluted 1:10 in nuclease-free water. Droplets were generated using the QX200 AutoDG Droplet Digital PCR System (BIO-RAD) with Automated Droplet Generation Oil for EvaGreen (#1864112, BIO-RAD). The miRNAs of interest [QIAGEN, mmu-miR-434–3p, #YP00205190, hsa-miR-183–5p, #YP00206030, hsa-miR-323a-3p, #YP00204278 (round 1); hsa-miR-9–3p, #YP00204620, hsa-miR-124–3p, #YP00206026, mmu-miR-212–3p, #YP00206022, hsa-miR-132–3p, #YP00206035 (round 2)] were analyzed on the same ddPCR plate, 11 (round 1) or 8 (round 2) samples per plate.

PCR was conducted by C1000 Touch^™^ Thermal Cycler (#1851196, BIO-RAD) with the following program: 95°C 5 min, 95°C 30 sec, 56 °C 1 min, repeat total 40 cycles, 4 °C 5 min, 90 °C 5 min, 4 °C hold. The droplets were quantified by the QX200 Droplet Reader (#1864003, BIO-RAD).

The ddPCR results were analyzed by QX Manager Standard Edition (version 1.1.341.0204, BIO-RAD).

#### Replicates and batch effect.

The plate contained 2 replicate wells for each sample. In addition, each plate contained an internal control sample to monitor possible differences between the ddPCR plates in each round. The sample was prepared from a TBI rat randomized into the UEF EpiBioS4Rx cohort (#1083) and sampled on D2, but was excluded from the follow-up due to FPI device malfunction.

#### Normalization miRNA levels to a reference gene.

The target miRNA concentrations were normalized to miR-28–3p concentration (target/reference) that was measured in each sample to obtain a normalized expression value for each sample.

#### Normalization of miRNA levels to exogenous control.

For ddPCR analysis of the UniSp6, cDNA was diluted 1:5000 in nuclease-free water. UniSp6 was measured by ddPCR from a total of 39 cDNA samples from 12 separate cDNA batches of the EpiBioS4Rx cohort (UniSp6 miRCURY LNA miRNA PCR Assay, #YP00203954, QIAGEN). The droplet generation, PCR, and droplet quantification were conducted as in the ddPCR analysis of EpiBioS4Rx samples.

### 2.9. Other factors

Analysis of the success of procedural harmonization among the three study sites was based on pre-defined CDEs recorded on Excel sheets. In addition, UEF performed additional experiments to evaluate the reproducibility of analysis methodologies. Successful harmonization of the TBI model has been reported previously (Ndode-Ekane et al., 2024b). Here, we focus on factors affecting the mRNA biomarker levels in plasma. As the analysis cohort was generated over the period of three years, we also investigated the practice effect on sampling and sample quality.

#### Housing conditions.

Details of housing conditions, possibly affecting the blood biomarker levels, are summarized in Table 1.

#### Changes in body weight D0-D2 (%).

Weight loss after severe TBI is well described (Lapinlampi et al., 2020). This apparently relates to brain injury-related impairment in consciousness, motor performance, and ongoing epileptiform activity (Andrade et al., 2019; Lapinlampi et al., 2020). Consequently, rats eat and drink less, particularly on D0-D3, but recover fast, starting on D3–4 when the drinking and eating behaviors resume.

To investigate the effect of weight loss on plasma miRNA levels on D2, rats were weighed before the induction of injury on D0 (injury day) and on D2 just before plasma sampling for miRNA analysis (Table 1). The analysis included the UEF and Monash animal sub-cohorts, as UCLA weighed the rats on D0 and D3. Weight change was calculated as [weight on D2/pre-injury weight on D0) * 100 %].

#### Volume correction.

Animals received 0.9 % NaCl twice a day for 3 days. On the day of blood sampling, saline was administered after the sampling.

### Duration of anesthesia during blood sampling on D2.

Anesthesia duration was defined as the time from the arrival of the rat in the anesthesia chamber to the withdrawal of the needle from the tail vein.

## 3. Results

Altogether, 245 rats (sham, TBI) were successfully epilepsy-phenotyped using 30-d vEEG and included in the final analysis cohort (Fig. 2). The ddPCR analysis was completed as planned in 85 % (209/245) of the cohort (Fig. 2). In UEF, miRNA ddPCR was performed in 91 % (91/100), in Monash in 87 % (73/84), and at UCLA in 74 % (45/61) of rats included in the final analysis cohort.

Next, we assess the factors that could have affected the (a) coverage of analysis in the study cohort and (b) miRNA levels within and between the three study sites.

### 3.1. Blood sampling and plasma processing

#### Labeling.

All samples used during the discovery and validation phases had proper labeling with sample-specific QR codes.

#### Time from impact to blood sampling on D2.

The average sampling time in the overall EpiBioS4Rx study was close to the aimed 48-h time point, being 46.6 h post-TBI in UEF and 47.8 h in Monash. The exact sampling time was not recorded at UCLA. In UEF and Monash combined, the largest deviation from the planned 48 h post-TBI time point was −7 to + 7 h.

#### Time used for plasma processing.

Plasma samples were prepared and flash frozen < 1 h of the blood withdrawal at all sites. The exact time was not recorded as a CDE and therefore, not systematically recorded.

### 3.2. Sample availability for miRNA analysis

#### Percentage of animals with successful blood sampling from the tail vein.

Of the 245 animals in the analysis cohort, D2 blood sampling was performed in 91 % (222/245) of rats. Site analysis indicated that sampling was performed from 92 % (92/100) of rats in UEF (35/43 MRI, 57/57 EEG), 93 % (78/84) of rats in Monash (35/41 MRI, 43/43 EEG) and 85 % (52/61) of rats in UCLA (28/37 MRI, 24/24 EEG) (Fig. 2B).

#### Volume of collected plasma samples.

According to the sample collection protocol for miRNA biomarker discovery and validation, plasma was stored in 50 μl aliquots (Fig. 3A_4_). The expected loss of sample volume during pipetting of the aliquots was < 3 μl. Consequently, we expected to get 47 μl of plasma from 4 aliquots shipped to UEF, resulting in a total sample volume of 188 μl for RNA extraction (Fig. 3B_1_). Aliquot volume < 47 μl were recorded and considered in further sample processing.

**Fig. 4.**
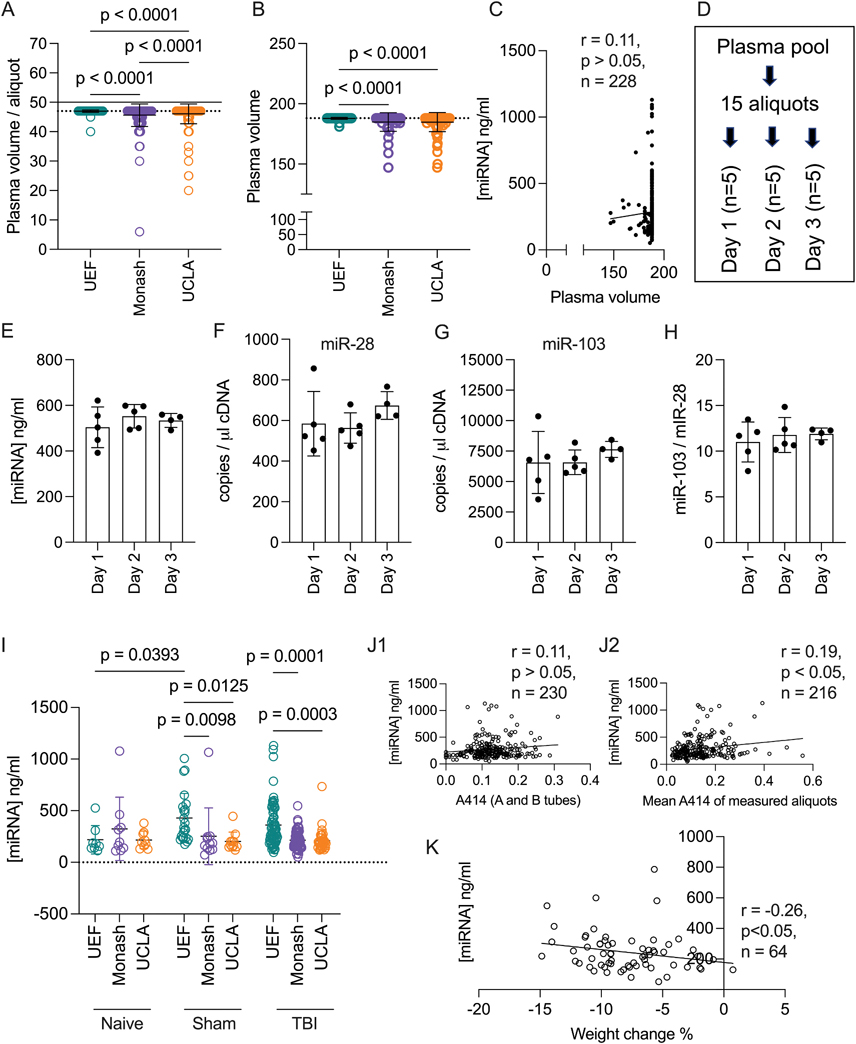
Pipetting accuracy and factors affecting total miRNA concentrations. (A) Volume of plasma shipped for miRNA assay was supposed to be 50 μl/vial, being enough for pipetting 47 μl for the small RNA extraction. The percentage of vials with plasma volume < 47 μl was 0.3 % (3/396) in UEF, 26 % (57/216) in Monash (p < 0.001 as compared to UEF, Kruskal-Wallis test followed by Dunn’s multiple comparisons test) and 14 % (45/328) in UCLA (p < 0.001 as compared to UEF or Monash). (**B**) Consequently, percentage of samples with total plasma volume < 188 μl required for small RNA extraction was 2 % (2/99) in UEF, 30 % (16/54) in Monash (p < 0.001 as compared to UEF) and 29 % (24/82) in UCLA (p < 0.001 as compared to UEF). (**C**) No correlation was found between the plasma volume and total miRNA concentration measured after the RNA extraction (p > 0.05). (**D**) To assess the effect of training of the researcher on the quality of RNA extraction, RNA was extracted and two miRNAs with high copy numbers (miR-28 and miR-103) were quantified using ddPCR in 15 samples pipetted from the same plasma pool over 3 d (5 extractions per day). By experience/training, standard deviation for **(E)** total miRNA concentration, **(F)** miR-28 copy number, **(G)** miR-103 copy number, and **(H)** miR-103/miR-28 ratio reduced between the analysis days. Next, we assessed the site effect on total miRNA concentration in naive, sham-operated and TBI animals. (**I**) **In the naive group** (baseline samples), total miRNA concentration did not differ between the three study sites (p > 0.05). **In the sham group**, the total miRNA concentration was higher in UEF than Monash (170 %, p < 0.001) or in UCLA (212 %, p < 0.05). **In the TBI group**, total miRNA concentration in UEF was higher than that in Monash (168 %, p < 0.001) or UCLA (172 %, p < 0.001). In UEF, the total miRNA concentration was higher in the sham than the naive group (196 %, p < 0.05). (**J1**) No correlation was observed between the hemolysis measured after plasma extraction (tubes A and B, Fig. 3A_**3**_) and the total miRNA concentration (r = 0.11, p > 0.05). (**J2**) There was a weak positive correlation between the A414 value in samples analyzed after pooling (Fig. 3B_**1**_) and the total miRNA concentration (r = 0.19, p < 0.05) (**K**) The greater the weight loss from D0 (injury day) to D2 (plasma sampling), the slightly higher the total miRNA concentration (r = −0.26, p = 0.0382) in the TBI group.

Altogether, in 11 % (105/940) of the plasma aliquots, the volume was < 47 μl. In UEF, 0.3 % (3/396) of aliquots had a volume < 47 μl (average sample volume 47 ± 0.4 μl)(Fig. 3A_4_, Fig. 4A). In Monash, 14 % (45/328) of aliquots had a smaller volume (46 ± 3 μl), which was higher than that in UEF (14 % vs. 0.3 %, p < 0.001) but lower than that in UCLA (14 % vs. 26 %, p < 0.001). In UCLA, 26 % (57/216) of aliquots had sample volumes < 47 μl (46 ± 4 μl), which was higher than that in UEF (26 % vs. 0.3 %, p < 0.001, Kruskal-Wallis followed by Dunn’s multiple comparisons test). In some vials, plasma volume was > 50 μl. To achieve the required sample volume for RNA extraction, the “extra” plasma in that tube was used to compensate for missing volume, if needed, and documented.

After pooling plasma from the 4 vials, 18 % (42/235) of samples had a volume < 188 μl. In UEF 2 % (2/99) of samples had a volume < 188 μl (average volume 188 ± 1 μl)(Fig. 3B_1_, Fig. 4B). In Monash, 29 % (24/82) of pooled plasma samples had a volume < 188 μl (185 ± 8 μl), which was higher than that in UEF (29 % vs. 2 %, p < 0.001). In UCLA, 30 % (16/54) of pooled plasma samples had a volume < 188 μl (185 ± 8 μl), which was higher than that in UEF (30 % vs. 2 %, p < 0.001).

In summary, 11 % of plasma aliquots were smaller than anticipated. However, most of the samples had only minor sample volume amounts missing when total volumes were compared. Importantly, there was no association between the plasma volume and extracted miRNA concentration (r = 0.11, p > 0.05, n = 228)(Fig. 4C), suggesting that a small (5–10 μl) difference in the starting material volume did not significantly affect the miRNA concentration obtained.

### 3.3. Hemolysis

**Fig. 5.**
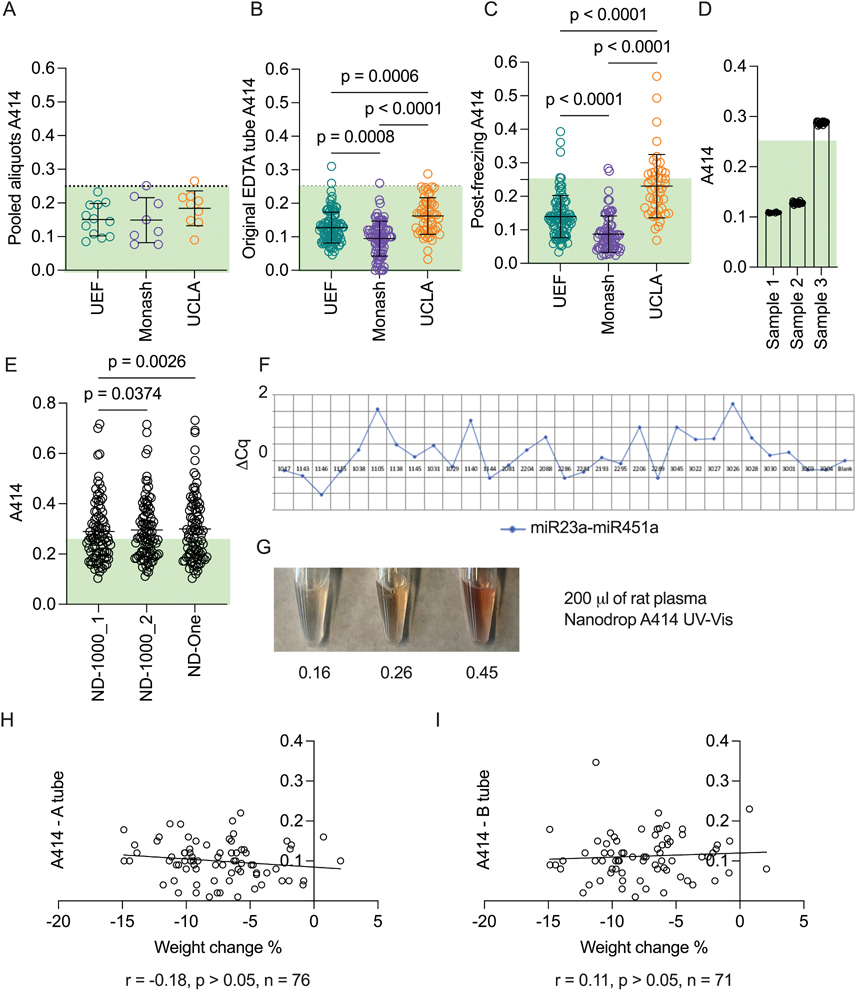
Plasma quality. (A) *Hemolysis in samples used for biomarker discovery*. Average A414 value of the thawed and pooled sample aliquots (Fig. 3B_1_) that were used for miRNA biomarker discovery (small RNA sequencing) did not differ between the study sites (12 samples from UEF, 8 Monash, 9 UCLA) (p > 0.05). Two samples had A414 values slightly > 0.25 (acceptance limit). (**B**) ***Hemolysis in samples used for biomarker validation*.** Average A414 value of plasma in the EDTA tubes (Fig. 3A_3_), from which plasma was aliquoted for validation of candidate miRNA biomarkers using droplet digital PCR (ddPCR) differed between UEF vs. Monash (p < 0.001), UEF vs. UCLA (p < 0.001), and Monash vs. UCLA (p < 0.001). Four samples had A414 values > 0.25. (**C**) Average A414 value of the thawed and pooled sample aliquots (Fig. 3B_1_) that were used for miRNA biomarker validation using ddPCR differed between UEF (104 samples) vs. Monash (82 samples)(p < 0.001), UEF vs. UCLA (54 samples)(p < 0.001) and Monash vs. UCLA (p < 0.001). Twenty-four plasma aliquots had A414 value > 0.25. (**D**) ***Reproducibility of A414 assay***. The A414 value of 3 samples from UEF tissue bank with different hemolysis levels (by eye) was measured 25 times (Nanodrop ND-1000_1, sample volume 1.5 μl). The average A414 values (mean ± standard deviation, range) for the 25 measurements were for Sample 1 0.109 ± 0.001 (range 0.106–0.112), Sample 2 0.128 ± 0.002 (range 0.123–0.133) and Sample 3 0.288 ± 0.003 (range 0.281–0.291), indicating high reproducibility of the assay. Thus, the variability in sample A414 values (panels B-C) related to variability in sample quality rather than to technical assay variability. (**E**) ***Comparison of spectrophotometers used for A414 assay.*** Spectrophotometer ND-1000_1 was used for hemolysis assay of all UEF EpiBios4Rx samples. When the same samples were analyzed using another ND-1000 spectrophotometer in UEF (ND-1000_2), the A414 values measured with ND-1000_2 were slightly higher than those obtained using ND-1000_1 (0.295 ± 0.122 vs. 0.289 ± 0.123, difference 0.006, p < 0.05). Also, A414 values analyzed using ND-One microvolume UV-Vis spectrophotometer were slightly higher than those obtained using ND-1000_1 (0.300 ± 0.134 vs. 0.289 ± 0.123, difference 0.010, p < 0.01). (**F**) ***dCq of miR-23a - miR-451 as hemolysis indicator.*** miR-23a – miR-451 (dCq) was < 2 in all samples used for miRNA discovery, indicating no hemolysis. Numbers in the panel refer to animal numbers – UEF samples start with 1, Monash with 2, UCLA with 3. Note that case #2088 was initially included in miR-sequencing but was later excluded from the analyses due to the short duration of phenotyping video-EEG on the 7th month. (**G**) Photographs showing the color of plasma samples with A414 value 0.16, 0.26, or 0.45. (**H-I**) ***Body weight and plasma quality*.** Decrease in body weight (%) did not correlate with increased A414 value either in the **(H)** A (r = −0.18, p > 0.05, n = 76) or **(I)** B tube (r = 0.11, p > 0.05, n = 71).

To quantify the severity of hemolysis, some of which was apparent on visual inspection of samples (Fig. 5), two assays were applied: absorbance at A414 nm and dCq miR-23a - miR-451 (van Vliet et al., 2017).

#### 3.3.1. Samples for miRNA biomarker discovery

##### EDTA tubes.

All plasma aliquots selected for miR-seq were from 30 samples that had A414 < 0.25 in tubes A/B measured on-site.

##### Thawed and pooled samples for miR-seq.

After shipping 30 frozen plasma samples to Qiagen Genomic services in Germany, hemolysis was measured again after thawing and pooling of aliquots before they were used for small RNA sequencing (Fig. 1B_1_). Of the 29 pooled aliquots included in the final analysis [1 of 30 samples (#2088) was excluded], two had A414 > 0.25 (0.251 and 0.265, Fig. 4A). Site analysis revealed that 0 % (0/12) of pooled samples had A414 above the acceptance limit (>0.25) in UEF, 13 % (1/8) in Monash and 11 % (1/9) in UCLA (Fig. 5A). The mean A414 value was 0.15 ± 0.05 (n = 12) in UEF, 0.15 ± 0.07 in Monash (n = 8) and 0.18 ± 0.05 in UCLA (n = 9) samples (p > 0.05 between the sites).

##### miR-23a vs. miR-451 delta (dCq).

miR-451 is a red blood cell-enriched miRNA and a marker of hemolysis (Shah et al., 2016). Therefore, the plasma samples sent for small RNA-seq underwent a hemolysis assessment using the miR-23a – miR-451 dCq method at Qiagen Genomic Services (Germany). The dCq was < 2 in all samples (n = 30), indicating no hemolysis (Fig. 5F).

#### 3.3.2. Samples for miRNA biomarker validation

##### EDTA tubes.

To investigate the hemolysis in the plasma aliquots that were used for ddPCR, we first analyzed A414 nm in the K2EDTA A/B tubes, of which the samples to ddPCR analysis were aliquoted (Fig. 3A_1_).

The average A414 value in UEF samples was 0.13 ± 0.05 (n = 104). In Monash, the average A414 value (n = 82) was 0.09 ± 0.05, being lower than in UEF (0.09 ± 0.05 vs. 0.13 ± 0.05, p < 0.001). In UCLA, the average A414 value was 0.16 ± 0.05 (n = 54), being greater than that in UEF (0.16 ± 0.05 vs. 0.13 ± 0.05, p < 0.001) or in Monash (0.16 ± 0.05 vs. 0.09 ± 0.05, p < 0.001) (Fig. 5B).

In a retrospective analysis performed after ddPCR, 4 samples included in ddPCR validation had A414 values above > 0.25 (0.26, 0.26, 0.29 and 0.31) in the K2EDTA tubes A/B (Fig. 5B).

##### Thawed and pooled samples for miR-seq.

Next, we investigated the A414 nm in all thawed plasma aliquots that had enough plasma (*i.e.,* ≥1.5 μl) available, after the 47-μl volume had been pipetted from the vial for RNA extraction. In UEF, 93 % (97/104), in Monash 88 % (72/82), and in UCLA 87 % (47/54) of samples had enough plasma left for the A414 measurement in at least one of the four 50-ul aliquots.

The average A414 value measured from the thawed aliquots (1–4 aliquots per sample) was 0.14 ± 0.06 for UEF (n = 97). In Monash (n = 72), the average A414 was 0.09 ± 0.05, being lower than that in UEF (0.09 ± 0.05 vs. 0.14 ± 0.06, p < 0.001). In UCLA (n = 47), the average A414 was 0.23 ± 0.09, being higher than that in UEF (0.23 ± 0.09 vs. 0.14 ± 0.06, p < 0.001) or in Monash (0.23 ± 0.09 vs. 0.09 ± 0.05, p < 0.001)(Fig. 5C). Of the 216 samples, 11 % (24/216) had an average A414 value > 0.25 (Fig. 5C).

### 3.4. Instrument-related variation in A414 assay

To better understand the difference between the A414 values in samples from the K2EDTA A/B tubes and samples from the aliquots that had been shipped to UEF, thawed, and pooled (Fig. 3A_3_ vs. Fig. 3B_1_), we performed two additional experiments.

#### Variability of A414 measurements – the same NanoDrop.

First, we measured A414 25 times in 3 plasma samples using the same NanoDrop equipment to assess methodological replicability. In the sample with A414 0.109, the SD of 25 measurements was 0.001 (range 0.106–0.112). In the sample with A414 0.128, the SD was 0.002 (range 0.123–0.133). In the sample with A414 0.288, the SD was 0.003 (range 0.281–0.291) (Fig. 5D). Note that plasma is often visibly pinkish when A414 value is > 0.25 (Fig. 5G).

#### Variability of A414 measurements – three different NanoDrops.

The A414 assay in K2EDTA tubes was performed on-site in UEF, Monash, or UCLA using the ND-1000 (NanoDrop^™^). In UEF, we next investigated whether the use of different equipment available at the site (labeled as ND-1000_1, ND-1000_2, ND-One) could affect the A414 level. ND-1000_1 was used in UEF assays throughout the EpiBios4Rx project. The average A414 of a sample analyzed with ND-1000_1 was slightly lower (<1 %) than that when analyzed using ND-1000_2 (0.289 ± 0.123 vs. 0.295 ± 0.122, p = 0.037)(Fig. 5E). The average A414 of the sample analyzed with ND-1000_1 was also slightly lower (4 %) than that analyzed using the ND-One (0.289 ± 0.124 vs. 0.300 ± 0.134, p = 0.003)(Fig. 5E).

Taken together, the UEF-site analysis revealed very mild variability between the A414 values measured using different spectrophotometers. The use of different equipment at different study sites was not considered to explain the differences in the A414 values observed between the original K2EDTA and post-freezing A414 tubes.

### 3.5. Effect of weight change and anesthesia duration on plasma hemolysis

Next, we analyzed if the anesthesia duration or reduction in body weight during the preceding 48 h of blood sampling affected the hemolysis.

#### Anesthesia duration during the D2 blood sampling.

Data are summarized in Fig. S1. In UEF, the anesthesia duration was 6.1 ± 2.1 min (range 3–13 min). In Monash, anesthesia duration was 7.6 ± 2.3 min (range 4–12 min), which was longer than that in UEF (7.6 ± 2.3 min vs. 6.1 ± 2.1 min, p < 0.001). In UCLA, the anesthesia duration was 4.2 ± 1.3 min (range 2–7 min), which was shorter than that in UEF (4.2 ± 1.3 min vs. 6.1 ± 2.1, p < 0.01) or Monash (4.2 ± 1.3 min vs. 7.6 ± 2.3 min, p < 0.001).

The longer the anesthesia duration, the lower the A414 value (n = 174, r = −0.20, p < 0.05).

#### Weight change.

Mean weight change observed between D0 and D2 was 10 ± 5 % (range 1–22 %). Weight change % did not correlate with A414 values either in the A (r = −0.18, p > 0.05) or B tube (r = 0.11, p > 0.05)(Fig. 5H-I).

### 3.6. miRNA analysis

#### 3.6.1. Training and efficiency of RNA extraction and ddPCR

To assess whether researcher training would improve the quality of the RNA extraction and ddPCR analysis, we performed RNA extraction and ddPCR analysis from a pooled plasma sample 15 times over a 3-d period (5 extractions per day, Fig. 4D).

##### RNA extraction.

Variability in extracted miRNA concentrations diminished over time, being 504 ± 89 ng/μl (range392 −621 ng/μl) on Day 1, 552 ± 52 ng/μl (range 494–598 ng/μl) on Day 2 and 534 ± 30 ng/μl (range 491–560 ng/μl) on Day 3 (Fig. 4E). The average concentration did not differ between days (p > 0.05). However, the variability reduced from Day 1 > Day 2 > Day 3. On Day 3, one sample had to be excluded due to an error in handling.

##### ddPCR.

Average copy number of miR-28 (endogenous control) was 584 ± 159 copies/μl on Day 1, 563 ± 75 copies/μl on Day 2 and 674 ± 68 copies/μl opn Day 3 (Fig. 4F). The average copy number of miR-103 (selected for its high copy number) was 6564 ± 2551 copies/μl on Day 1, 6581 ± 1010 copies/μl on Day 2 and 7644 ± 660 copies/μl on Day 3 (Fig. 4G). The average copy number did not differ between days (miR-28 and miR-103, both p > 0.05). In both cases, the variability reduced from Day 1 > Day 2 > Day 3. Also, when miR-103 levels were normalized to miR-28 levels, variability decreased from Day 1 (11.0 ± 2.2) > Day 2 (11.8 ± 1.9) > Day 3 (11.9 ± 0.6) (Fig. 4H). The normalized miR-103 levels did not differ between the analysis days (p > 0.05).

In summary, researcher-related variability in RNA extraction and ddPCR analysis can be reduced by training. In the EpiBioS4Rx biomarker study, all RNA extractions were performed by one experienced technician (M.L.) and ddPCR analyses by one trained PhD student (M.H.).

#### 3.6.2. Total miRNA concentration – effect of study site, hemolysis and weight loss

##### 3.6.2.1. Study site.

###### Naive.

Total miRNA concentration in plasma was similar between the study sites (all comparisons p > 0.05, Fig. 4I).

###### Sham-operated experimental controls.

In UEF, the average total miRNA concentration was 429 ± 238 ng/ml (n = 23). In Monash, the average total miRNA concentration was 253 ± 275 ng/ml (n = 11), which was 59 % of that in UEF (p < 0.01). In UCLA, the average total miRNA concentration was 202 ± 91 ng/ml (n = 11), which was 47 % of that in UEF (p < 0.05) and 80 % of that in Monash (p > 0.05)( Fig. 4I).

Interestingly, in UEF, the average total miRNA concentration in the sham group was 196 % of that in the naive group (p < 0.05)(Fig. 4I). In Monash or UCLA, no differences were observed in miRNA concentration between the experimental groups (p > 0.05).

###### TBI.

In UEF, the average total miRNA concentration was 360 ± 217 (n = 68). In Monash, the average total miRNA concentration was 214 ± 89 (n = 62), which was 59 % of that in UEF (p < 0.01). In UCLA, the average total miRNA concentration was 210 ± 109 (n = 34), which was 58 % of that in UEF (p < 0.001) and 98 % of that in Monash (p > 0.05)( Fig. 4I).

##### 3.6.2.2. Hemolysis.

A414 value in the K2EDTA tubes did not correlate with the miRNA concentration (r = 0.11, p > 0.05, n = 230)(Fig. 4J1).

However, the greater the A414 value in thawed and pooled samples, the greater the miRNA concentration (r = 0.19, p < 0.05, n = 216, Fig. 4J2). We do not consider this to explain the site differences in miRNA concentration, as samples from UEF with the highest miRNA concentration had no significant hemolysis detected.

##### 3.6.2.3. Weight change.

Greater weight decrease from D0 to D2 was associated with higher miRNA concentrations (r = −0.26, p = 0.0382, n = 64, Fig. 4K).

### 3.7. Assessment of miRNA expression profile in EpiBioS4Rx plasma samples

#### 3.7.1. Read counts in different treatment groups and study sites

**Fig. 6.**
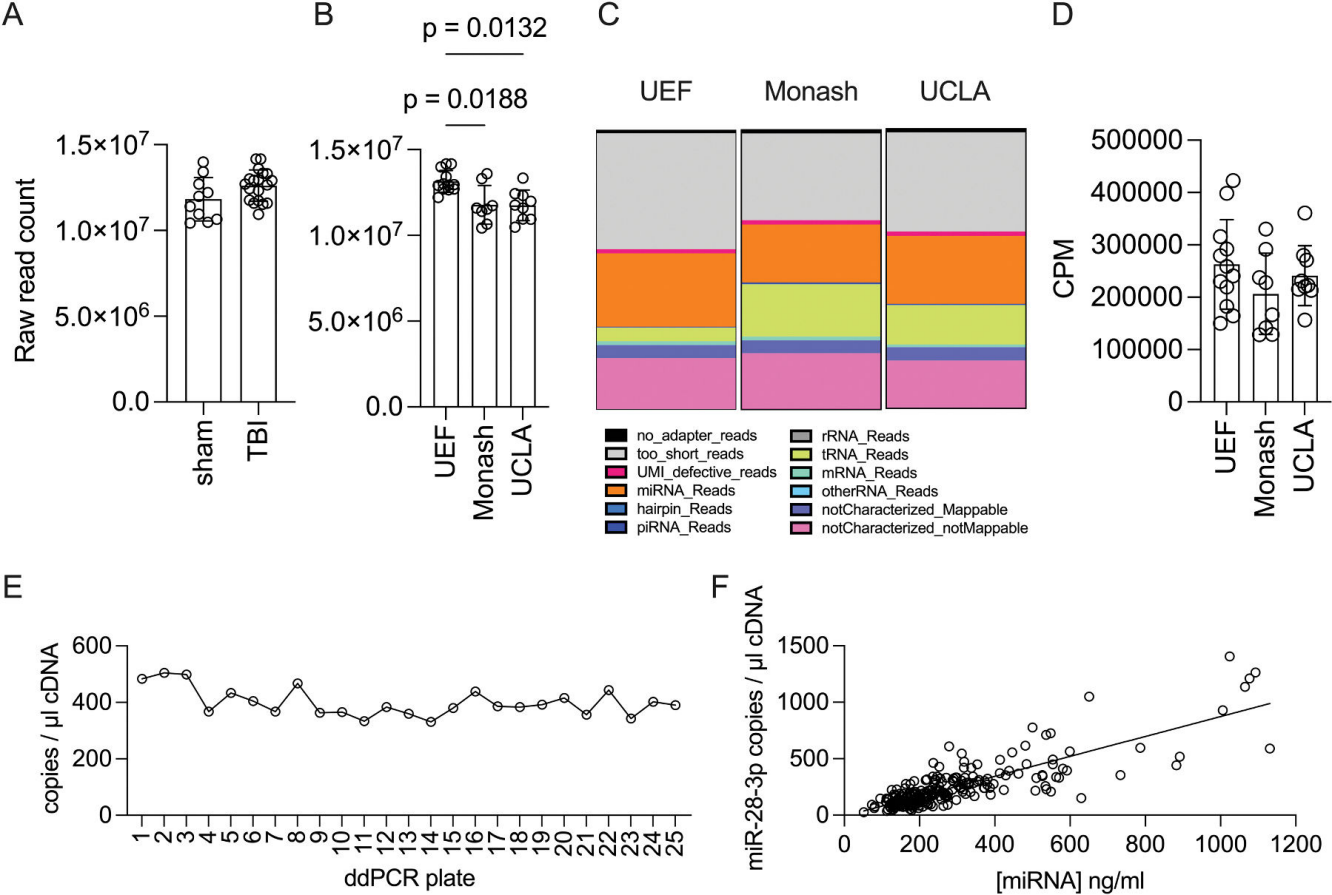
Small RNA quality. (A) The number of small RNA raw reads in plasma samples used for miRNA discovery did not differ between the sham-operated experimental controls (n = 10) and rats with TBI (n = 19). (**B**) In UEF samples (sham and TBI combined, n = 12), the raw read count was slightly higher than that in Monash (n = 8, p < 0.05) or UCLA (n = 9, p < 0.05). (**C-D**) Even though there was a slight difference in the raw read counts (CPM) between the sites (panel D), the number of small RNAs in different RNA categories normalized to total read count did not differ between the study sites (p > 0.05). (**E**) miR-434 used as a control for day-to-day variability in miRNA levels between the plates, as it has shown consistent expression in our samples (about 400 copies/μl). Consequently, its level was measured from the same sample in all PCR 25 plates analyzed on Round 1 (see text). (**F**) miR-28–3p was used as a stable endogenous control. The greater the total miRNA concentration, the higher the miR-28–3p copy number (n = 235, r = 0.790, p < 0.001, 95 %CI 0.74–0.84).

The raw read counts in samples used for miRNA biomarker discovery did not differ between the sham and TBI groups (all animals within the group combined) (Fig. 6A), However, the read counts (naive, sham, TBI combined) in UEF were 112 % (n = 12) of that in Monash (13 152 880 ± 639039 vs. 11 744 307 ± 1064934, p < 0.05, Fig. 6B) and 112 % of that in UCLA (13 152 880 ± 639039 vs. 11 756 631 ± 889012, p < 0.05, Fig. 6B).

The number of miRNA molecules detected belonging to different miRNA categories was rather similar between the study sites (Fig. 6C). After normalizing the miRNA counts to total read counts (counts per million reads), no differences were detected between the sites (p > 0.05, Fig. 6D).

Based on findings at discovery phase, we conclude that miRNA profiles in animals generated at different study sites were comparable.

#### 3.7.2. Control miRNAs and normalization

Based on our previous experiments and method optimization (Das Gupta et al., 2020), ddPCR protocol was set-up with commercial miRCURY LNA primers (Qiagen) for the miRNAs of interest (miR-183–5p, miR-434–3p, miR-323–3p, miR-9a-3p, miR-124–3p, miR-132–3p and miR-212–3p).

##### cDNA synthesis - exogenous and endogenous controls.

To control variance caused by the cDNA synthesis, Qiagen Genomic Services measured exogenous controls UniSp6, UniSp-100 and UniSp-101 prior to sequencing. These spike-ins had similar profiles in all samples from UEF, Monash and UCLA (Fig. S2A). However, experiments at UEF showed variable results between cDNA synthesis batches (i.e., synthesis days, 1 batch/d) when UniSp6 was used as an exogeneous control (Batch 4 vs. 5, p < 0.05, Batch 4 vs. 7, p < 0.05, Batch 4 vs. 10, p < 0.05, Fig. S2B). No difference between the days (batches) was found when endogenous control miR-28–3p was used to control the success of DNA extraction (Fig. S2C). Further, no correlation was found between UniSp6 and miR-28–3p levels (r = 0.01, p > 0.05, n = 38, Fig. S2D).

Next, we transcribed the same sample to cDNA four times on the same day. miR-28–3p produced more consistent results than UniSp6 (Fig. S2E1–3). Consequently, we concluded that using the endogenous control miR-28–3p gave more stable results than using the exogenous control UniSp6. Consequently, miRR-28–3p was used for data normalization in ddPCR.

##### ddPCR normalization - miR-28–3p.

For each 7 miRNA biomarker candidate, miR-28–3p levels were assessed on the same ddPCR plate. As shown above, miR-28–3p levels correlate with miRNA concentration (Fig. 6F) and its levels remained stable over different cDNA synthesis reactions (Fig. S2, **panel E3**). To mitigate the assay variability, miR-28–3p measured on the same PCR plate as the biomarker candidates was used for normalization, that is, to calculate the relative concentration of each miRNA.

##### ddPCR plate control.

To monitor reproducibility between the ddPCR runs/plates, miR-434–3p concentration was analyzed (TBI D2 plasma) on all ddPCR plates processed during Round 1 of the validation phase (25 plates). miR-434–3p levels ranged from 331 to 505 copies/μl (Fig. 6E).

#### 3.7.3. Body weight changes

As the miRNA concentration in the TBI samples were higher than that in the sham or naive samples, we investigated whether weight loss from D0 to D2 (and smaller plasma volume) was associated with miRNA levels.

##### Effect on reference miR-28–3p.

The higher the level of reference miR-28–3p, the greater the weight change % in both Round 1 (r = −0.26, p = 0.0395, n = 64, Fig. S3A) and Round 2 (r = −0.27, p = 0.0309, n = 64, Fig. S3B), even though the correlations were weak.

##### Effect on candidate biomarker miRNAs.

Next, we investigated whether the weight change % correlated with the levels of the 7 other miRNAs that were validated with ddPCR. We found that the concentration (copies/μl cDNA) of miR-434–3p (r = −0.28, p = 0.0259, n = 64, Fig. S4A), miR-183–5p (r = −0.38, p = 0.0020, n = 64, Fig. S4C), miR-212–3p (r = −0.30, p = 0.0143, n = 64, Fig. S4K) and miR-132–3p (r = −0.30, p = 0.0167, n = 64, Fig. S3M) weakly correlated with weight change % at day 2 after surgery.

After normalization to miR-28–3p, only miR-183–5p showed a slight negative correlation with weight change % (r = −0.25, p = 0.0482, n = 64, Fig. S4D).

Other measured miRNAs (miR-323–3p, miR-9a-3p and miR-124–3p) did not correlate with weight change % before or after normalization (Fig. S4).

#### 3.7.4. Stability of miRNA in plasma samples

**Fig. 7.**
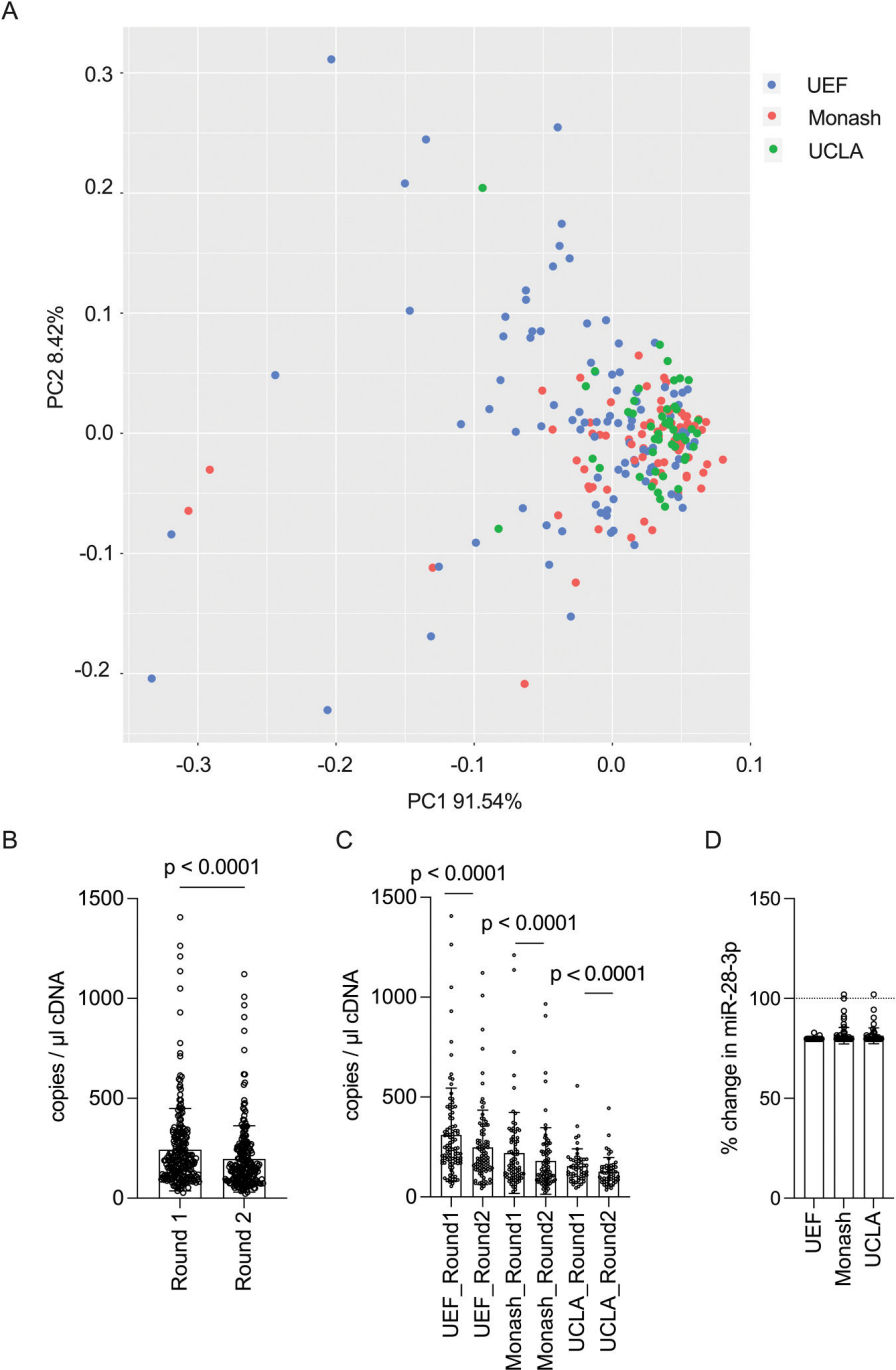
miRNA analysis pipeline standardization. (A) Principal component analysis (PCA) of measured sample parameters (A414, plasma volume, and miRNA concentration) revealed similar rather than different patterns in samples between the study sites when principal component 1 (PC1) and 2 (PC2) were plotted. (**B**) Using the same sample as in Round 1, miR-28–3p (used as a stable endogenous control) was also measured in Round 2 (see text)(228 samples). The time interval between Rounds 1 and 2 was 7–12 months. miR-28–3p concentration on Round 2 was 81 % of that on Round 1 (p < 0.001). (**C-D**) Reduction in miR-28–3p levels from Round 1 to Round 2 was rather similar between the study sites (UEF reduction 20 ± 0.4 %, n = 96; Monash 19 ± 4 %, n = 79; UCLA 19 ± 4 %, n = 53; p < 0.001 for all). **(E)**. The decrease in miR-28–3p concentration from Round 1 to Round 2 was comparable at all study sites.

The ddPCR analyses of the EpiBioS4Rx were performed on two rounds (3 miRNAs in Round 1 and 4 miRNAs in Round 2) with one year apart. miR-28–3p was measured on both rounds on the same PCR plate as the candidate miRNA biomarkers. Consequently, we were able to compare the miR-28–3p concentrations between the rounds. Interestingly, miR-28–3p concentration on Round 2 was 19 ± 3 % lower than on Round 1 (n = 228, Fig. 7B). Decrease in miR-28–3p concentration was systematic across all study sites (20 ± 0.4 % decrease in UEF, n = 96; 19 ± 4 % decrease in Monash, n = 79; 19 ± 4 % decrease in UCLA, n = 53; all p < 0.001 as compared to Round 1; Fig. 7D).

Overall, principal component analysis revealed that samples from different study sites used for the ddPCR were similar rather than different (Fig. 7A).

## 4. Discussion

Despite the increasing number of preclinical multicenter studies aiming at biomarker discovery for neurological diseases, only a handful of studies have attempted prospective, rigorous harmonization of study protocols and standardization of methodologies across multiple participating centres (Kamnaksh et al., 2019; Kochanek et al., 2016; Lapinlampi et al., 2017; Ndode-Ekane et al., 2024b; Santorelli et al., 2024; van Vliet et al., 2017; Wanner et al., 2025). Our objective in the multicenter EpiBioS4Rx Project 1 was not only to perform a harmonized study but also to assess the success of procedural harmonization and methodological standardization in plasma miRNA biomarker discovery. We had five major findings. First, miRNA levels were successfully measured in 85 % of the 245 rats included in the final analysis cohort. Second, the major factors resulting in missing data were small sample volume, hemolysis, and failed RNA extraction for ddPCR. Third, the use of endogenous control produced more stable results than using exogenous spike-ins. Fourth, anesthesia duration and weight change had minor effects on hemolysis and/or total miRNA levels. Fifth, the training of researchers improved data reproducibility. We also identified gaps in our processes, especially related to data recording and training of the personnel.

### 4.1. Issues related to blood collection and processing affecting circulating miRNA biomarker analysis

The planned time point of blood sampling for circulating biomarker analysis was 48 h after TBI or sham operation, which was based on preliminary experiments and review of miRNA and protein biomarker literature in rodent models of TBI (Ndode-Ekane et al., 2024b). We also had to appreciate the regulatory issues limiting the sample volume. Only < 2.0 ml of blood per week can be drawn from an adult 400 g rat, limiting the number of biomarkers analyzed in each sample as well as sample re-analysis (https://nc3rs.org.uk; https://oacu.oir.nih.gov).

The average delay from injury to sampling was close to the aimed time point of 48 h, being 46.6 h post-TBI in UEF and 47.8 h post-TBI in Monash. In UCLA, the exact sampling time was not recorded, but it was around 48 h. In UEF and Monash combined, the largest deviation from the planned 48 h post-TBI time point was −7 to + 7 h. We considered the timing of sampling accuracy acceptable for plasma biomarker analysis and did not consider deviations as an exclusion criterion, as the discovery approach was omics-based, and therefore, the expression kinetics of specific candidate biomarkers could not be investigated in advance. However, the secretion kinetics of the candidate biomarkers is an issue to consider before deciding on the sampling time point.

As isoflurane anesthesia used during TBI/sham surgery and blood sampling can have neuroprotective effects in brain injury models and antiepileptogenic efficacy in a rat model of temporal lobe epilepsy, we attempted to standardize the length of the anesthesia as closely as possible across the study sites to avoid its confounding effects on epileptogenesis (Bar-Klein et al., 2016; Jiang et al., 2017; Neag et al., 2020; Statler et al., 2006; Sun et al., 2015). Even though there were minor inter-site differences in the duration of isoflurane anesthesia during blood sampling on D2 (or baseline), the few-minute difference was not considered as a criterion for sample exclusion, as the total anesthesia duration mostly derived from the TBI surgery on D0 (Immonen et al., 2019; Ndode-Ekane et al., 2024b). Interestingly, despite longer anaesthesia in the EEG than the MRI cohort, miRNA levels tended to be higher in the EEG than the MRI cohort, probably related to electrode-implantation-induced brain injury (Heiskanen et al., 2024).

According to the sampling protocol, centrifugation of blood tubes and preparation and flash-freezing of plasma samples had to be within 1 h of blood withdrawal. However, as the time from blood sampling to freezing was not included in the CDEs, the success in harmonization could not be evaluated retrospectively.

Transportation of plasma samples from Monash and UCLA to UEF and from UEF to Qiagen Genomic Services in Germany was conducted by a biopharmaceuticals courier service in a temperature-controlled dry ice shipping package to ensure that the samples were maintained at a low temperature. When the samples arrived at the final location, personnel receiving the package confirmed that the samples were indeed frozen, and the package had enough dry ice left. We did not observe any systematic differences between the samples originating from different study sites that could be due to the sample shipment or storage.

Only a small portion of the plasma samples were discarded on site due to poor plasma quality (A414 >0.25), being 4 % of samples in UEF, 1 % in Monash, and 14 % in UCLA. We also assessed hemolysis during the discovery phase after thawing and pooling sample aliquots from 30 rats before sending them to Qiagen Genomic Services. No differences in the mean A414 values were observed between the sites. However, two samples had A414 values > 0.25. This motivated us to perform a thorough assessment of post-thawing hemolysis for all > 200 samples included in ddPCR validation. Altogether, 24 samples had A414 > 0.25, which could not be explained by analytical factors such as equipment or methodologies used. Rather, we assume that red cell contamination is related to suboptimal pipetting, that is, a small quantity of the cellular pellet ended up in the pipette tip during pipetting of plasma aliquots after centrifugation. Also, previous miRNA studies have emphasized proper pipetting as a critical step to prevent plasma contamination by the cellular pellet after the centrifugation step (Das Gupta et al., 2020; Kim et al., 2017; Kroh et al., 2010). Moreover, in 4 cases with A414 above the threshold limit that had been included in the ddPCR analysis, there was a mistake in data entry to the Excel sheet in two cases, typing error in one case, and a single failure to notice that a value was above the acceptance limit, indicating human error. In future studies, inclusion of a second check of hemolysis values or automation of the sample selection pipeline could reduce human errors.

We also observed discrepancies in the volumes of aliquoted plasma. Of 940 aliquots, 11 % contained less than the required 47 μl plasma for miRNA analysis. We also observed a few samples with volumes greater than 50 μl. Interestingly, the number of incomplete aliquots differed between the study sites, varying between 0.3 % and 26 %. This could be due to incorrect calibration of pipettes or pipetting technique, particularly as multiple researchers were involved in plasma aliquoting. These observations underline the importance of proper training and the need for self-evaluation of pipetting accuracy.

### 4.2. Issues related to RNA processing and miRNA analysis affecting the biomarker analysis

Screening of potential biomarker candidates by small RNA sequencing was performed at Qiagen Genomic Services. Despite some inter-site differences in the raw read counts, the sequencing results in samples from different sites were similar. Furthermore, the list of detected miRNAs was almost identical between sites (Heiskanen et al., 2024). As we already knew that the lateral FPI model production had been well-harmonized and the epilepsy outcome was comparable between sites (Ndode-Ekane et al., 2023), the similarity in small RNA sequencing data in plasma samples of a subpopulation of rats encouraged us to proceed to the laborious validation phase.

The selection of methods for small RNA sequencing and downstream analysis of the sequencing data affects the workload and the output (Androvic et al., 2022; Bisgin et al., 2018; Wong et al., 2019). Small RNA profiling by next-generation sequencing can be affected, for example, by interference from RNA modifications (Munafó and Robb, 2010), biases in library amplification (Buschmann et al., 2016; Dabney and Meyer, 2012; Sendler et al., 2011), methods to measure abundance (Zyprych-Walczak et al., 2015), or the amount of input RNA required for the library construction (Head et al., 2014; Lopez et al., 2015). It is important to acknowledge that not all potential biomarker candidates may be discovered by using only one discovery technique, particularly if the sample size is low. Use of information in public databases like SRA (www.ncbi.nlm.nih.gov/sra) and in other open-access resources supports the decision on the final selection of miRNA candidates for validation. We were alerted to issues related to small RNA -seq after we found differences in EpiBioS4Rx and EPITARGET sequencing data (Heiskanen et al., 2024, 2023; van Vliet et al., 2024). For example, in EpiBioS4Rx but not in EPITARGET, plasma miR-9a-3p, miR-183–5p, and miR-434–3p were regulated after TBI. In EPITARGET but not in EpiBioS4Rx, miR-124–3p, miR-132–3p, and miR-212–3p were differentially expressed. miR-323–3p was differentially expressed in both cohorts. As no samples were analyzed using both RNA-seq platforms, we can only speculate on the possible reasons for the differences. These include (a) a greater heterogeneity in the EpiBios4Rx (multicenter) than EPITARGET (single-center) animal cohort, (b) use of different RNA isolation kits (even though both from Qiagen), (c) use of different RNA-seq library kits, which were available at the time of sequencing (EpiBioS4Rx 2019–2020, QIAseq miRNA library Kit from Qiagen; EPITARGET 2017, TruSeq Small RNA Library preparation kit from Illumina)(differences reviewed by Benesova et al. (2021), and (d) difference in sequencing depth (EpiBioS4Rx 12.3 M, EPITARGET 24.7 M). Importantly, ddPCR validation demonstrated that all 7 miRNAs included in the validation were robustly upregulated in TBI rats in EpiBioS4Rx samples (Heiskanen et al., 2024).

After screening, we chose to validate biomarker candidates by ddPCR to obtain interindividual variations and assess the true potential of selected miRNAs as biomarkers for epileptogenesis. We chose reagents and kits for ddPCR validation from multiple vendors. We first had to verify their functionality prior to running the validation phase. Moreover, optimization of ddPCR assay parameters such as the annealing temperature or cycle number can reduce the number of intermediate droplets (also called “droplet rain”) resulting in better separation of positive and negative droplets and improving the quality of results (Jones et al., 2014; Witte et al., 2016). To optimize the ddPCR method, we adjusted the temperature gradient for each miRNA assay. Consequently, all selected biomarker candidates were validated (Heiskanen et al., 2024).

The ddPCR validation of potential biomarkers was conducted at one study site (UEF) by one researcher (M.H.) to minimize technical variability. To monitor variation between ddPCR runs, we included a plate control sample in each run and measured its miR-434–3p levels. As expected, we found some batch-to-batch variation. Therefore, we measured the levels of stable endogenous reference, miR-28–3p, in each sample to enable normalization of each miRNA expression to reference miRNA. Therefore, the slight variability observed between the ddPCR runs did not affect the results.

Several methods exist for gene expression normalization in ddPCR experiments. Since ddPCR measures absolute quantity, it does not necessarily require the use of a reference gene as in RT-qPCR (Campomenosi et al., 2016; Hindson et al., 2013). For example, one approach for normalization is to fix the volume or RNA concentration of the samples before ddPCR (Das Gupta et al., 2020; Kang et al., 2022; Tavano et al., 2018). There are also exogenous synthetic RNA spike-ins that can be added to the sample to monitor the efficacy of RNA extraction and cDNA synthesis (Blondal et al., 2013; Faraldi et al., 2019; Vigneron et al., 2016). On the other hand, normalization to endogenous reference(s) is also a valid approach (Gupta et al., 2021; Hayden et al., 2024; Heiskanen et al., 2023; Hsu et al., 2024; Karttunen et al., 2024). It is crucial to select a reference gene (or multiple references) that is stable across the samples from different experimental groups, meaning that its expression is not affected by the disease condition (Donati et al., 2019)). Unfortunately, there are still no standard references for preclinical or clinical miRNA biomarker studies, and therefore, the references used vary from study to study. In the EpiBioS4Rx study, we selected miR-28–3p as the endogenous reference gene for normalization, as it was found to be stably expressed by the NormFinder analysis, and we have also used it in our previous studies assessing samples from the same TBI model (Gupta et al., 2021; Heiskanen et al., 2023). The use of miR-28–3p was also supported by our observation in EpiBioS4Rx samples that miR-28–3p levels strongly correlated with the miRNA concentration.

RNA samples are typically stored frozen either at −20 °C or −80 °C or under liquid nitrogen. In the present study, plasma samples were stored at −70°C before ddPCR analysis. The storage time from sampling to ddPCR analysis was up to 4 years, as the animal experiments were performed in sub-cohorts over 3 years. This raised a question about whether the integrity of RNA was maintained during storage as some ribozymes can maintain activity even at −70 °C (Fabre et al., 2014). Previous studies have demonstrated that miRNAs are highly stable in frozen plasma and can remain unchanged for several years (Balzano et al., 2015; Matias-Garcia et al., 2020). Therefore, we concluded that the preprocessing storage at −70°C did not influence the miRNA analysis.

After thawing the samples, RNA was extracted from all samples in one batch. However, due to the large sample number, the miRNA ddPCR analyses were conducted in two batches, one year apart. Assessment of the reference miRNA, miR-28–3p, concentration indicated that the second thawing of the extracted RNA (batch 2) that was stored in −70 °C for 1 year resulted in an average of 19 % decrease in its levels. Even though extracted miRNA samples have been demonstrated to remain stable in −70 or −80 °C for at least 1 year (Mraz et al., 2009; Sourvinou et al., 2013) and instructions given by the commercial manufacturer of the RNA extraction kit suggest one year stability (www.qiagen.com/us/resources/faq?id=d28e72d8-ee39-41ea-8eb8-222ffec4007e&lang=en), repetitive freeze-thawing of plasma can reduce miRNA concentration (Glinge et al., 2017). We assume that the reduction in miR-28–3p levels between analysis rounds is related to the additional freeze-thaw cycle of extracted RNA rather than the storage time. Since miR-28–3p was the only miRNA that was measured in both rounds, it remains to be studied whether some reduction occurred in the concentrations of other miRNAs. However, as the individual miRNAs from all animals were analyzed in the same batch, the additional thawing of extracted RNA was not considered to affect the biomarker results.

### 4.3. Other factors affecting miRNA concentrations in preclinical

Genetics, sex, housing conditions, feeding, post-injury weight loss, and pre-sampling drug-treatment could affect plasma miRNA concentrations (Enright et al., 2018), and therefore, the EpiBioS4Rx study sites made a major effort to harmonize these aspects (Ndode-Ekane et al., 2024b). All centers used male Spraque-Dawley rats, although from different vendors. Before the FPI surgery, the rats in UEF were single-housed, whereas at other sites they were housed in groups of 2–3 rats. After FPI surgery, rats had comparable volume replacement and analgesic treatment and were single-housed under similar conditions at all study sites. Unlike at other sites, in UCLA, post-surgery treatment also included antibiotics. Interestingly, we found that the greater the post-surgery weight loss, the higher the miRNA concentration.

### 4.4. Towards optimizing the preclinical biomarker discovery studies for clinical translation

Our study focused on miRNA biomarker discovery in a rat model to guide the assessment of clinical samples. Several issues should be considered to improve translation. First, multi-center preclinical studies should follow predetermined standard operating procedures for sample collection, processing, and miRNA analysis to control technical bias. Additionally, a strategy to address protocol deviations should be established. Second, resources for continuous monitoring and training of procedures during the project duration should be budgeted. Third, preliminary studies should determine the optimal time for blood sampling relative to the expression and duration of presence of miRNA in plasma after TBI, which may vary by miRNA and differ between rodents and humans. Fourth, animal studies should be blinded, statistically powered, and matched to anticipated clinical biomarker discovery relative to sex and variability in the time of day of sampling. Also, studies should consider the effect of feeding and the drugs used (including type and duration of anesthesia) on miRNA levels. Fifth, preclinical studies should include naïve animals instead of (or in addition to) sham-operated experimental controls as a reference to assessing the magnitude and direction (increase, decrease) of changes in miRNA levels to avoid, for example, craniotomy or electrode-implantation -induced changes in miRNA expression. Sixth, there should be a pre-determined strategy for data analysis, validation of target miRNAs found using miRNA-seq, as well as validation of biomarker findings in an independent animal cohort.

## 5. Conclusion

The present analysis highlights the importance of procedural harmonization between laboratories, protocol standardization, inclusion and analysis of quality controls, researcher training, and continuous monitoring of adherence to pre-agreed protocols.

## Supplementary Material

1

## Figures and Tables

**Table 1:** Rats, housing, surgery, post-operative care, and blood sampling at the three study sites for miRNA biomarker discovery and validation.

	UEF	Monash	UCLA

**Animals and housing** Number of rats randomized	184	194	147
Strain and species	Spraque-Dawley rats	Spraque-Dawley rats	Spraque-Dawley rats
Vendor (Country)	Envigo Laboratories B. V. (The Netherlands)	In-house breeding (Australia)	Charles River (USA)
Sex	Male	Male	Male
Food pellets	2016S (Teklan Diet) (Envigo Laboratories B.V., The Netherlands)	102108 (Barastoc, Australia)	LabDiet 5001* (LabDiet, St. Loius, MO, USA)
Duration of quarantine	7 days	3–7 days	At least 3 days
Housing before impact surgery	Group of 1 rat	2–3 rats	Group of 2 rats
Lights-on/lights-off cycle	7.00 am light-on/7.00 pm lights-off	7.00 am light-on/7.00 pm lights-off at Monash University and 6.00 am light-on/6.00 pm lights- off at the Melbourne University	6:00 am light-on/6:00 pm lights-off
Room temperature (C)	22 ± 1 °C	22 ± 1 °C	20–26 °C
**Surgery and post-operative care (D0-D2)**			
Weight at the time of injury	354 ± 18 g (range 315 – 408)	349 ± 39 g (range 250 – 440 g)	336 ± 41 g (range 260 – 497 g)
Anesthesia system	Somnosuite #SS6069B (Kent Scientific)	Somnosuite #SS6069B (Kent Scientific)	Matrix VIP 3000 Vaporizer #91305430 (Patterson Veterinary)
Anesthetic	Isoflurane (5 % induction & 2 % maintenance)	Isoflurane (5 % induction & 2 % maintenance)	Isoflurane (5 % induction & 2 % maintenance)
Medical Oxygen	Not applicable	Mediquip Medical Equipment & Supplies, Australia	Not applicable
Analgesia	Buprenorphine (Orion Pharma, Finland)	Buprenorphine (Indivior Pty Ltd, Australia)	Flunixin meglumine (MERK, USA)
Other treatments	None	None	Trimethoprim sulfamethoxazole (TMS) medicated rodent chow, Envigo Laboratories, USA (3 days after injury)
Additional feeding	Powdered pellets	Milk powder, mixed with powdered pellet and water provided ad libitum, until rats recovered their pre-injury weight	None
Housing after impact surgery	Single-housed	Single-housed	Single-housed
**Blood sampling from lateral tail vein (D2)** (https://nc3rs.org.uk/3rs-resources/blood-sampling/blood-sampling-rat; Van Vliet et al. 2017; Kamnaksh et al. 2019)			
Weight on D2	Sham	Sham	Sham
	TBI	TBI	TBI*
Timing of sampling	8:00–14:00	8:00–16:00	10:00–16:00
Anesthesia system	Somnosuite #SS6069B	Somnosuite #SS6069B	Matrix VIP 3000 Vaporizer #91305430
	(Kent Scientific)	(Kent Scientific)	(Patterson Veterinary)
Timing of baseline sampling	4–7 d before TBI/sham operation	Usually, 3–7d before TBI. On some occasions, it was closer to day of TBI and in 3 cases on same day as TBI.	3–14 d before TBI/sham operation
Anesthetic	Isoflurane 5 % for induction and 1–1.9 % for maintenance	Isoflurane 5 % for induction 1.5–2 % for maintenance	Isoflurane 5 % for induction and 1.5–2 % for maintenance
Medical Oxygen	Not applicable	Yes	Not applicable
Analgesia	Not applicable	Not applicable	Flunixin meglumine (MERK, USA)
Fluid supplementation	Saline (i.p., 30 ml/kg, b.i.d.) on D0-D1, on D2 after blood sampling 60 ml/kg/ 24 h, D3 60 ml/kg/24 h	Saline (i.p.) on D2 after blood sampling (10 ml/ kg, 2–3 times/day until recovery)	Saline (i.p., 30 ml/kg) on D0 after injury and D2 after blood sampling
Anesthesia duration during blood sampling (induction and sampling)	~2–3 min	~3–5 min	~3 min (1.5–2 %)
Needle	23 G butterfly (#367284, BD Vacutainer)	23 G butterfly needle (#367284, BD Vacutainer)	23 G butterfly needle (#367284, BD Vacutainer)
Tube A & B (temperature)	500 μl K2EDTA-tube (#365974, Vacutainer, BD Biosciences, Franklin Lakes, NJ, USA) (on ice)	500 μl K2EDTA-tube (#365974, Vacutainer, BD Biosciences, Franklin Lakes, NJ, USA) (on ice)	500 μl K2EDTA-tube (#365974, Vacutainer, BD Biosciences, Franklin Lakes, NJ, USA) (on ice)
Time to centrifugation Centrifuge (#), speed, temperature, duration	~20 min Eppendorf Biotools centrifuge Model 5417 R (1300 g, 10 min, +4 °C)	~30 min Eppendorf Biotools centrifuge Model 5417 R (1300 g, 10 min, +4 °C)	~20 min Eppendorf Biotools centrifuge Model 5415 R (1300 g, 10 min, +4 °C)
Eppendorf vials	Eppendorf^™^ Protein LoBind^™^ Tubes (022431064)	Eppendorf^™^ Protein LoBind^™^ Tubes (022431064)	Eppendorf^™^ Protein LoBind^™^ Tubes (022431064)
Hemolysis measurements	ND-1000 (NanoDrop^™^)	ND-1000 (NanoDrop^™^)	ND-1000 (NanoDrop^™^)
Storage temperature	−70° C	−70° C	−70° C
Number of investigators that performed the sampling over the three years	1	< 3	<5
**Shipping of plasma samples**			
Storage time before shipping**	1st sample: 3/2017 Last sample: 9/2019 No shipping (on-site analysis)	1st sample: 7/2017 Last sample: 5/2019 Shipping to Finland: 4/2021	1st sample: 2/2018 Last sample: 11/2019 Shipping to Finland: 04/2021.
Shipping temperature	Not applicable	−70° C	−70° C

*Abbreviations:* D, day; K2EDTA, di-potassium ethylenediaminetetraacetic acid; TBI, traumatic brain injury; *, weighted on day 1 and day 3. **Droplet digital polymerase chain reaction analysis: round 1 between May 2021 to August 2021 and round 2 between March 2022 to May 2022. See text for details.

## Data Availability

The data that support the findings of this study are available on request from the corresponding author.

## Appendix A. Supporting information

Supplementary data associated with this article can be found in the online version at doi:10.1016/j.eplepsyres.2025.107667.
