# Supplementary material for: Standardization of preclinical methodologies for discovery and validation of circulating microRNA biomarkers for post-traumatic epileptogenesis – Lessons learned from the EpiBioS4Rx Project 1
Source: Epilepsy Res. Author manuscript; Available in PMC 2026 Jun 15. (PMC13265265; doi:10.1016/j.eplepsyres.2025.107667)
Supplement: 1 [file NIHMS2183321-supplement-1.docx]

**Appendix 1: Supporting information**

**Supplementary Figures S1-4**


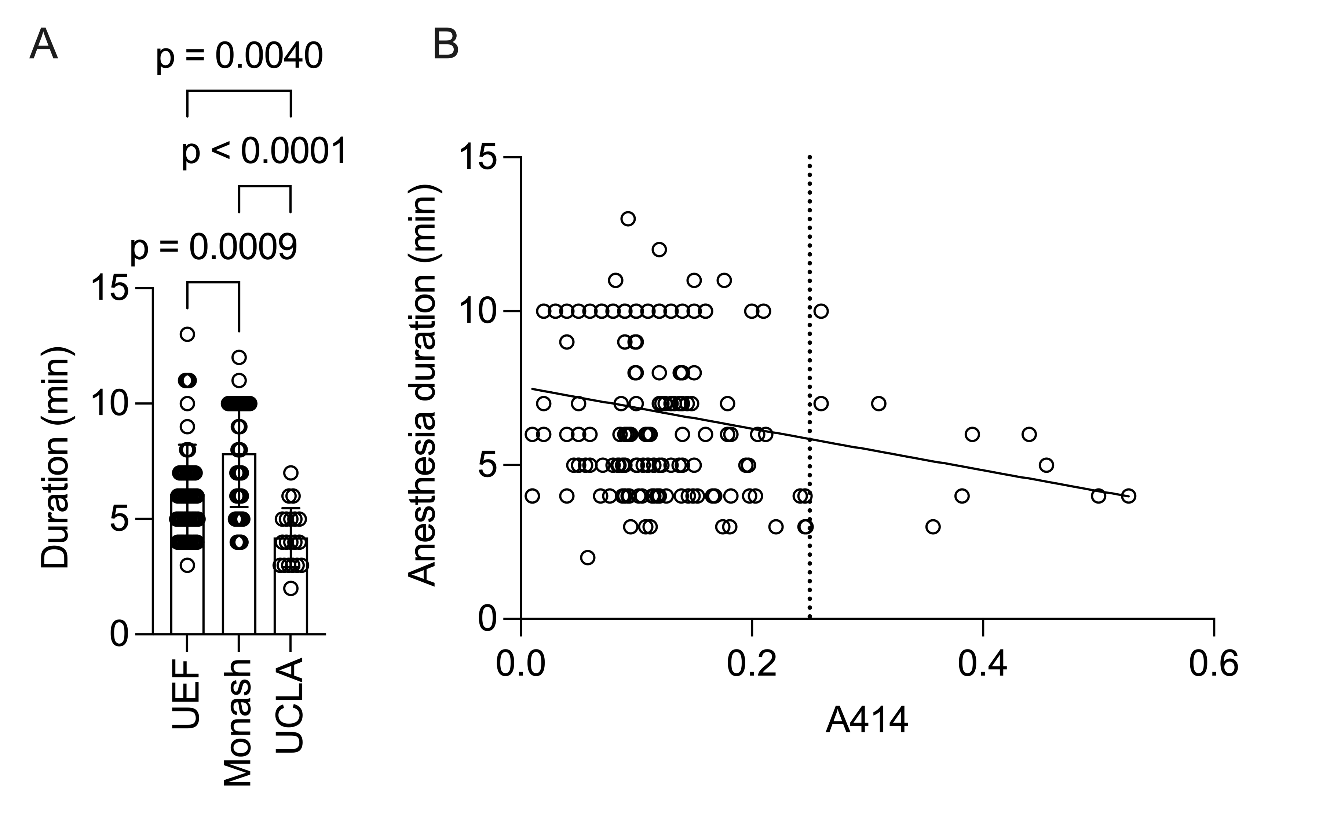


**Figure S1.** ***Duration of the isoflurane anesthesia during blood sampling on D2.*** (**A**) In UEF, the duration of anesthesia induced for blood sampling on D2 was 6.1 ± 2.1 min. In Monash, the anesthesia duration was 7.9 ±2.3 min, being longer than that in UEF (UEF vs. Monash, p<0.001). In UCLA, the anesthesia duration was 4.2 ± 1.3, being shorter than that in UEF (UCLA vs. UEF, p<0.01) or in Monash (UCLA vs. Monash, p<0.001). **(B)** The smaller the A414 value (indicator of hemolysis), the longer the anesthesia duration (n=174, r = -0.20, p <0.05). Note that the samples with the poorest quality (A414 >0.25, dashed line) had the shortest anesthesia duration.

**
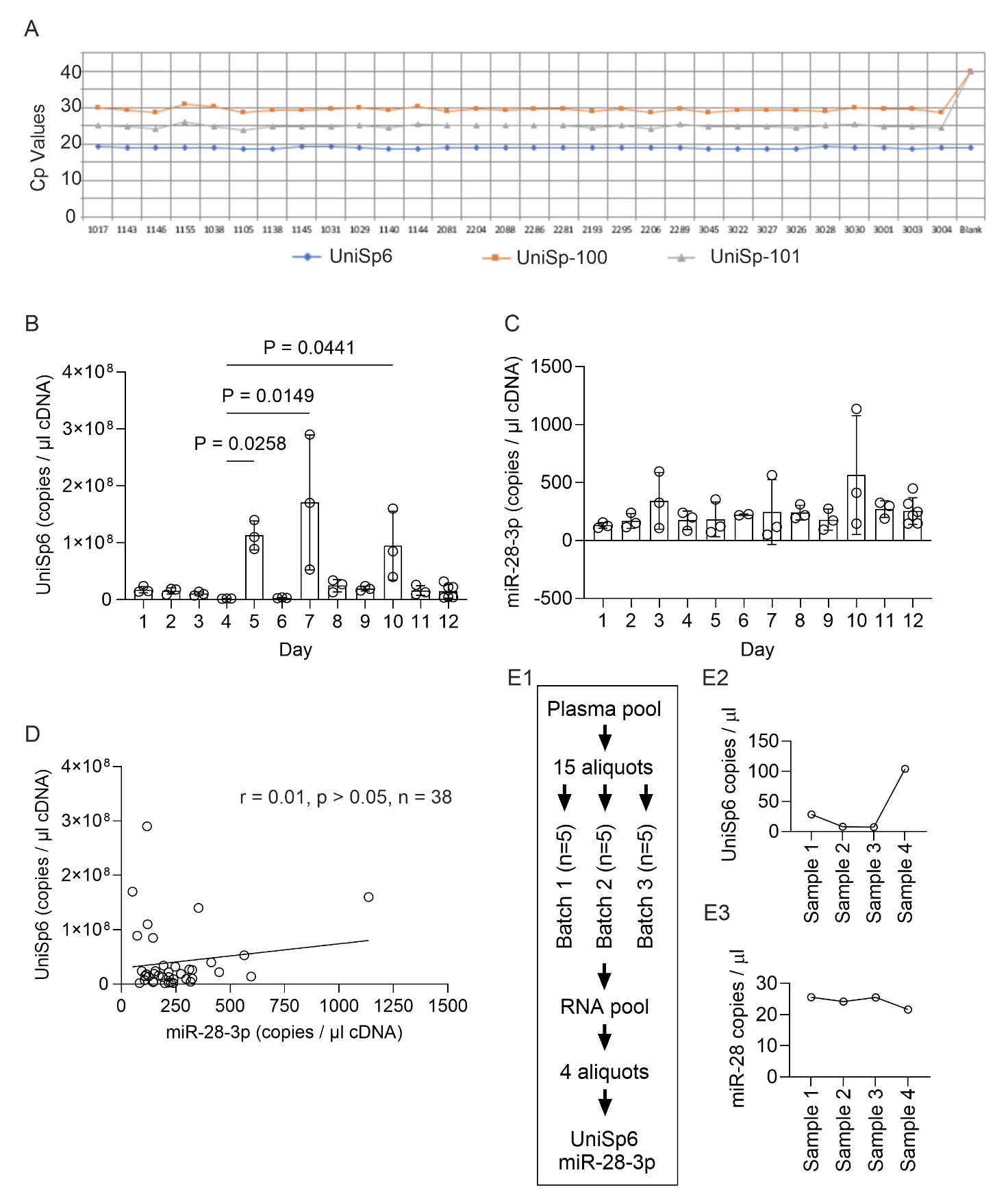
**

**Figure S2.** ***cDNA synthesis quality*.** (**A**) Qiagen Genomic Services measured UniSp6, UniSp-100, and UniSp-101 as external controls for sample quality before miRNA-sequencing in 30 samples initially sent for miRNA discovery (note that sample #2088 was excluded at the later analysis phase). Marker profiles were comparable in all samples from different study sites (UEF samples 1017-1144, Monash 2081-2289 and UCLA 3045-3004). In UEF samples, (**B**) UniSp6 copy numbers varied, depending on the cDNA synthesis day (day 4 vs. day 5, p<0.05; day 4 vs. day 7, p<0.05; day 4 vs. day 10, p< 0.05). (**C**) Unlike UNiSp6 levels, mir-28-3p copy numbers did not differ between the cDNA synthesis days. (**D**) also, UniSp6 and miR-28-3p levels did not correlate (n=38, r=0.01, p>0.05). (**E1-3**) To compare UniSP6 and miR-28-3p as “stable” internal standards, **(E1)** a plasma pool was prepared from samples in the UEF tissue bank. Then, on the same day, four aliquots from the pooled sample were transcribed to cDNA. **(E2)** UniSp6 levels were more variable than **(E3)** miR-28-3p levels.

**
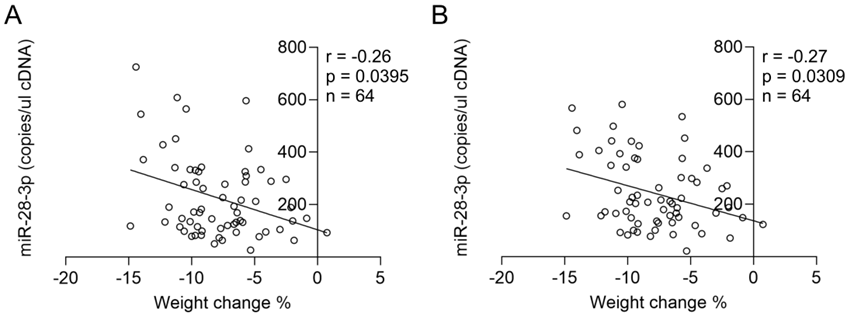
**

**Figure S3.** ***Levels of internal standard*** ***miR-28-3p and change in body weight*.** The greater the post-TBI reduction in body weight (%), the higher the miR-28-3p levels on (**A**) Round 1 (n=64, r = -0.26, p<0.05) and (**B**) Round 2 (n=64, r = -0.27, p<0.05).

**
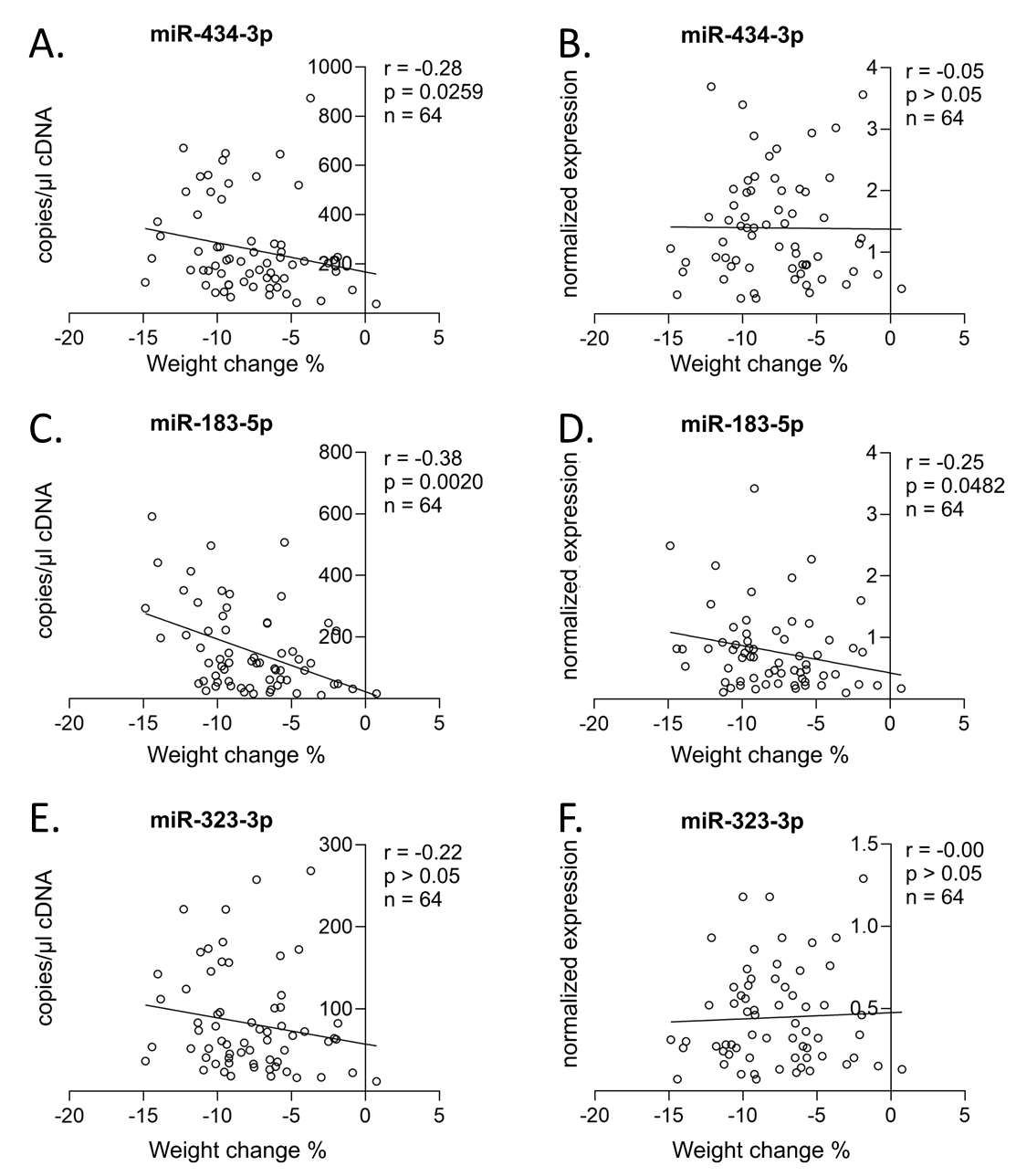
**

**
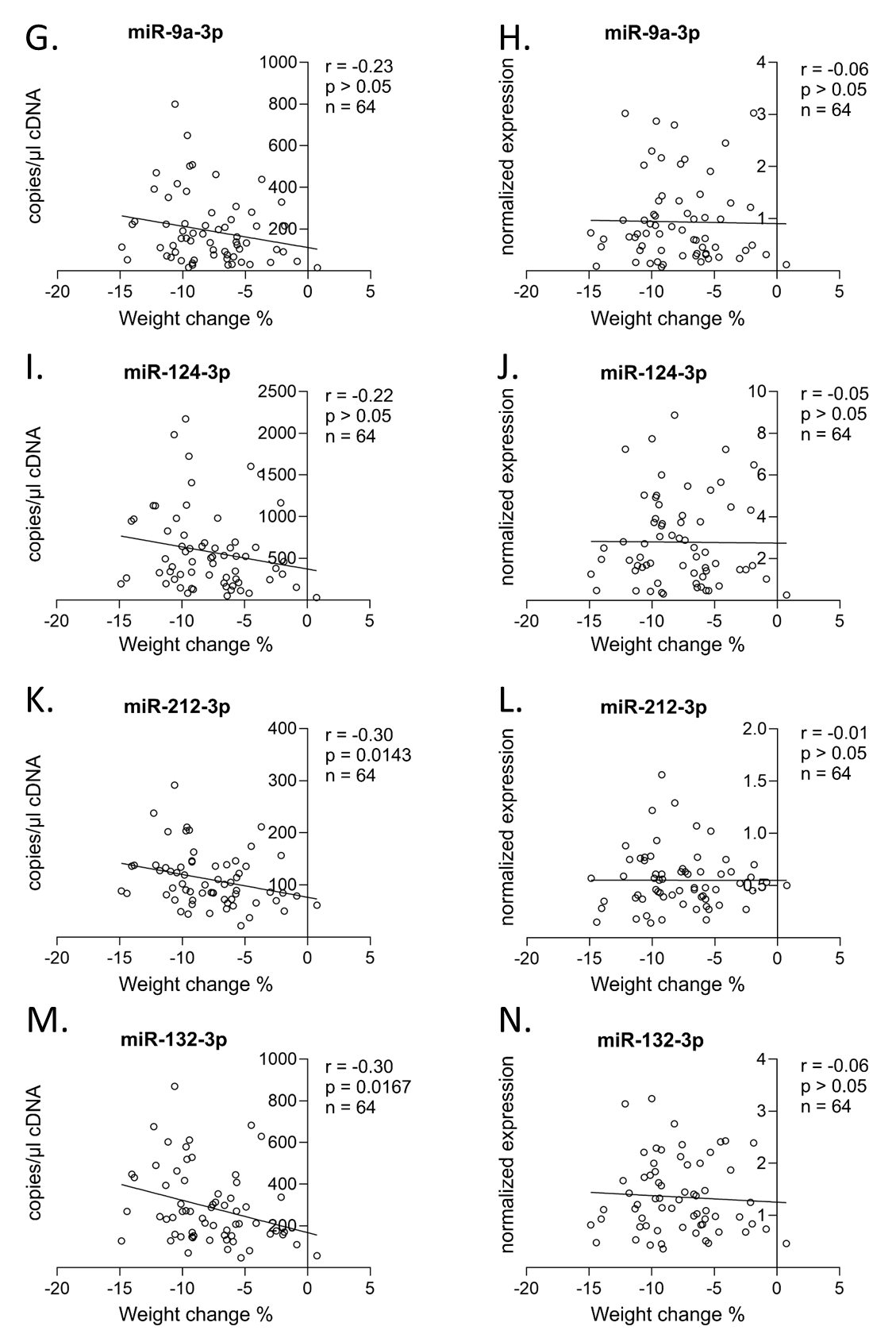
Figure S4.** ***Levels of biomarker candidates miR-434-3p, miR-183-5p and miR-323-3p and change in body weight.*** **(A**) The greater the miR-434-3p copy number, the greater the weight reduction (%) in the TBI group (n=64, r = -0.28, p<0.05). **(B)** No association was found between the miR-434-3p levels and body weight loss after data normalization to miR-28-3p. **(C)** The greater the miR-183-5p copy number, the greater the weight reduction (%) in the TBI group (n=64, r = -0.38, p<0.01). **(D)** The association between miR-183-5p and weight loss survived the normalization to miR-28-3p levels (n=64, r = -0.25, p<0.05). (**E-F**) miR-323-3p levels did not correlate with the weight change before or after data normalization. **(G-H)** miR-9a-3p and **(I-J)** miR-124-3p did not correlate with weight change before or after the data normalization. (**K**) The greater the copy number of miR-212-3p, the greater the weight loss (n=64, r = -0.30, p<0.05). **(L)** No association was found between the miR-212-3p levels and body weight loss after data normalization to miR-28-3p. **(M)** The greater the copy number of miR-132-3p, the greater the weight loss (n=64, r = -0.30, p = 0.0167). **(N)** No association was found between the miR-132-3p levels and body weight loss after data normalization to miR-28-3p.
